# Exploring tetrazole chemistry: synthetic techniques, structure–activity relationship, and pharmacological insights in antimicrobial and anticancer therapy

**DOI:** 10.3389/fchem.2025.1700143

**Published:** 2025-12-08

**Authors:** Lalmohan Maji, Ghanshyam Teli, Rohit Pal, Neelesh Maheshwari, Praveen Kumar Soni, Gurubasavaraja Swamy Purawarga Matada, Mahendra Singh Rathore, Venkatesan Saravanan, Kathiravan Muthukumaradoss

**Affiliations:** 1 Department of Pharmaceutical Chemistry, SRM College of Pharmacy, Faculty of Medicine and Health Sciences, SRM Institute of Science and Technology, Chengalpattu, Tamil Nadu, India; 2 School of Pharmacy, Sangam University, Bhilwara, Rajasthan, India; 3 Geetanjali Institute of Pharmacy, Geetanjali University, Udaipur, Rajasthan, India; 4 Integrated Drug Discovery Center, Department of Pharmaceutical Chemistry, Acharya and BM Reddy College of Pharmacy, Bengaluru, Karnataka, India; 5 School of Pharmacy, SBV Chennai, Sri Balaji Vidyapeeth (Deemed to be University), Pondicherry, India; 6 Dr. A. P. J. Abdul Kalam Research Lab, Department of Pharmaceutical Chemistry, SRM College of Pharmacy, Faculty of Medicine and Health Sciences, SRM Institute of Science and Technology, Chengalpattu, Tamil Nadu, India

**Keywords:** tetrazole, anticancer, antimicrobial, synthetic strategy, structure–activity relationships

## Abstract

Tetrazoles are nitrogen-rich heterocycles that have attracted interest because of their numerous applications in pharmaceutical and medicinal chemistry. Four nitrogen atoms and one carbon atom make up these five-membered rings, which have special physicochemical and electrical characteristics, including acidity, resonance stabilization, and aromaticity. This article highlights the structure, spectroscopic characteristics, and physical and chemical characteristics of tetrazoles. It also describes how overlapping mechanisms, such as DNA replication inhibition, protein synthesis disruption, and oxidative stress induction, as well as similar therapeutic targets, enable inhibitors to serve as both antibacterial and anticancer agents. Tetrazole moieties have been fused with a range of pharmacophores, such as indoles, pyrazoles, quinolines, and pyrimidines, yielding fused derivatives that display substantial inhibitory activity against bacterial, fungal, and cancer cell lines, with certain compounds exhibiting efficacy comparable to or exceeding that of established therapeutic agents. The rational design of more efficacious tetrazole-based therapies is facilitated by structure–activity relationship analysis, which further highlights significant functional groups and scaffolds that contribute to increasing activity. We investigate the relationship between microbial inhibition and anticancer efficacy, opening up new avenues for the creation of multifunctional therapeutic agents. We hope that this study will offer significant guidance and serve as a valued resource for medicinal and organic researchers working on drug development and discovery in multifunctional therapeutics. The review involves a thorough investigation of tetrazole in recent years.

## 1 Introduction

### 1.1 Structure and spectroscopic properties

**FIGURE 1:**
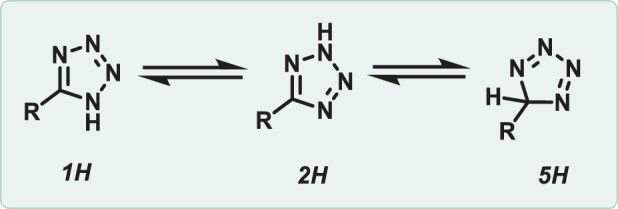
Tautomers of tetrazole.

**FIGURE 2:**
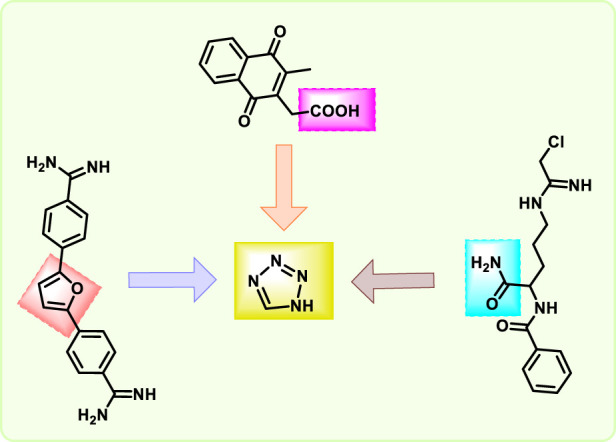
Structure and bioisostere analog of tetrazole.

Tetrazoles are an instance of a heterocyclic compound that has a five-membered ring with one carbon atom and four nitrogen atoms. This unique arrangement makes them highly nitrogen-rich, leading to special chemical and energetic properties. Tetrazoles can theoretically exist in three tautomeric forms, like 1H, 2H, and 5H (Figure 1). Among these, the 1H and 2H tautomers of tetrazole are aromatic, each possessing a 6π-electron system that contributes to their stabilization. On the other hand, the 5H tautomer is not aromatic and is merely a proposed theoretical structure that lacks experimental proof (Kiselev et al., 2011). Tetrazole is found in the 1H form in the solid state, and the 1H tautomer predominates in polar solvents like dimethylformamide (DMF) and dimethyl sulfoxide (DMSO). However, in the gas phase, the 2H tautomer is more prevalent (Bugalho et al., 2001). The tetrazole ring can act as a bioisostere for carboxylic acid groups (Biot et al., 2004), Cl-amidine rings (Subramanian et al., 2015), and furan rings in drug molecules because they share similar structural and electronic properties (Herr, 2002; Mohite et al., 2009) (Figure 2). Frequently, it enhances the metabolic stability and ADMET profile of compounds containing these functionalities. In drug design, tetrazoles can proficiently emulate carboxylic acids due to their comparable electronic distribution and hydrogen-bonding properties (Allen et al., 2012; Ballatore et al., 2013).

**FIGURE 3:**
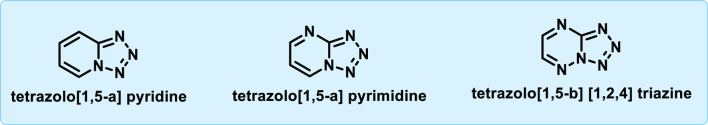
Some examples of substituted tetrazole bicycles fused with pyridine, diazine, or triazine.

The two most significant isomers in terms of pharmacology and synthesis are 1*H*-tetrazole and 2*H*-tetrazole. The proton’s location on the nitrogen atom distinguishes the tautomer 1*H*- and 2*H*-tetrazoles (Uppadhayay et al., 2022). These two forms are tautomers, distinguished by the position of a proton on the nitrogen atom. Factors such as solvent polarity, pH, and temperature affect their dynamic equilibrium in solution. They remain thermodynamically stable in the solid state. This technique enhances both the thermodynamic stability and the aromatic character of the compound. The proton is on the N2 nitrogen in 2*H*-tetrazole, making it frequently more stable in polar liquids. Numerous kinds of bicyclic tetrazole systems have been developed through combining the tetrazole ring with heterocycles like pyridine, diazine, or triazine. Notable examples include tetrazolo [1,5-*a*]pyridine, tetrazolo [1,5-*a*]pyrimidine, and tetrazolo [1,5-*b*][1,2,4]triazine (Figure 3).

Tetrazoles exhibit distinctive infrared absorption bands linked to different ring vibrations and specific functional groups. The N–H stretching band of 1*H*-tetrazole commonly shows up between 3,150 cm^−1^ and 3,400 cm^−1^. C=N stretching takes place in the 1600–1,500 cm^−1^ region, while N=N stretches are observed between 1,400 cm^−1^ and 1,300 cm^−1^. Moreover, in the range between 800 cm^−1^ and 1,000 cm^−1^, ring deformation and out-of-plane N–H bending vibrations take place (Billes et al., 2000). Tetrazoles exhibit prominent π→π* electronic transitions, often occurring at 210–230 nm in the ultraviolet spectrum. This technique is beneficial for exploring electronic effects and ring substitution. Modification of the tetrazole ring can lead to bathochromic changes, also known as red shifts, due to enhanced conjugation. Additionally, 1- and 2-substituted tetrazoles can be differentiated by their distinct UV absorption spectra. For example, 1-phenyltetrazole absorbs light at 236 nm, while 2-phenyltetrazole absorbs light at 250 nm. In contrast, NH-unsubstituted tetrazoles often exhibit altered absorption due to significant intermolecular interactions (Popova et al., 2017). Nuclear magnetic resonance (NMR) in both one-dimensional (^1^H, ^13^C, ^15^N) and two-dimensional (COSY, HMBC, and HSQC) forms is one of the most effective methods for determining tetrazole structures and identifying regioisomers and tautomers (Shestakova et al., 2013). The ^1^H NMR method was used to characterize the free N–H bond in tetrazole. In ^1^H NMR spectroscopy, tetrazole had a peak downfield and a pKa value comparable to that of carboxylic acid. One signal was observed in ^13^C NMR spectroscopy at 155–160 ppm, depending on the substituted tetrazole ring. Tetrazole’s proton is highly aromatic and has the capacity to stabilize π-electron delocalization in a very acidic manner. The tetrazole anion is lipophilic, which is a crucial property for medication development (Elewa et al., 2020; Khurshid et al., 2022). Mass spectrometry of tetrazoles reveals distinct fragmentation behaviors in both positive and negative ion modes. In the positive ion mode, the molecule typically loses HN_3_, while in the negative ion mode, it tends to lose N_2_. As a result, these different ionization conditions lead to characteristic and differing fragmentation patterns during analysis (Liu et al., 2008).

### 1.2 Physical and chemical properties of tetrazoles

Tetrazole appears as a white to pale yellow crystalline powder with no detectable odor. It has a melting point of 155–157 °C, a density of 1.477 g/cm^3^, and a molar mass of 70.05 g/mol (Uppadhayay et al., 2022). Tetrazole is soluble in water, ethyl acetate, DMSO, and DMF. Tetrazole behaves as a weak acid with a pKa of approximately 4.89, comparable to propanoic acid (Jaiswal et al., 2024). Its acidic character arises from the pyridine-like nitrogen atom in the heteroaromatic ring, which enables delocalization of negative charge and coordination with metal ions. The substituent electrostatic properties strongly influence the acidity of 5-substituted tetrazoles; for example, 5-phenyltetrazole exhibits acidity similar to benzoic acid due to resonance stabilization of its conjugate base. Tetrazole anions readily form upon reaction with metal hydroxides and remain stable in aqueous or alcoholic media, even at elevated temperatures.

Chemically, tetrazoles react vigorously with strong acids, acid chlorides, anhydrides, oxidizing agents, and certain active metals, producing heat, toxic fumes, or explosive products. Upon heating or combustion, they decompose and release carbon monoxide, carbon dioxide, and nitrogen oxides. In biochemical contexts, dilute 1*H*-tetrazole in acetonitrile is widely used as an activating agent in oligonucleotide synthesis. The tetrazole moiety functions as a carboxyl group bioisostere, improving pharmacokinetic profiles by enhancing solubility and bioavailability while reducing adverse effects. It also exhibits both electron-withdrawing (–I) and electron-donating (+M) effects, rendering tetrazole derivatives versatile in supramolecular chemistry and coordination complexes (Bissantz et al., 2010; Meyer et al., 2003; Movsisyan et al., 2016; Uppadhayay et al., 2022; Varala and Babu, 2018).

Pharmacologically, tetrazole-containing compounds display diverse biological activities, including antibacterial (Roszkowski et al., 2021), antifungal, anticancer (Slack et al., 2011), antiviral, anti-inflammatory, analgesic, antidiabetic, antihyperlipidemic, antitubercular, antinociceptive, and hypoglycemic effects (Nammalwar et al., 2015). Prominent therapeutic applications include treatments for hypertension (Belz et al., 1999), allergies, infections, and seizures. Consequently, tetrazole and its derivatives are of significant interest across medicinal chemistry, biochemistry, and agricultural research.

### 1.3 Synthetic methods of tetrazole formation

#### 1.3.1 From amine

**FIGURE 4:**
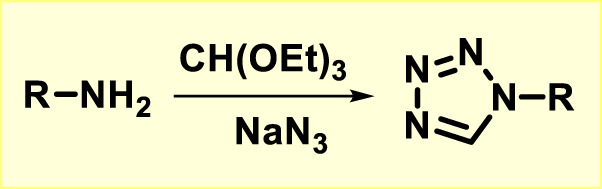
Synthesis of tetrazole from amine.

The most basic method for synthesizing 1-substituted tetrazoles involves the reaction of an amine molecule with triethyl orthoformate in the presence of sodium azide using DMSO (Figure 4) (Dighe et al., 2009; Palimkar et al., 2003).

#### 1.3.2 From cyano compounds

**FIGURE 5:**
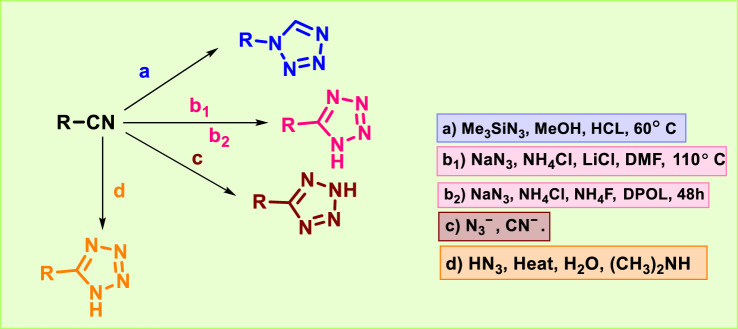
Synthetic route of tetrazoles from cyano compounds.

Nitriles or isocyanides are used as starting materials in a well-known and efficient approach for synthesizing substituted tetrazoles (Figure 5). 1-Substituted tetrazole was synthesized by reacting isocyanide with trimethylsilyl azide (1.5 eq) in 0.5 M methanol under acidic conditions at 60 °C (a) (Jin et al., 2004). Nitrile was treated with sodium azide, ammonium chloride, and lithium chloride in anhydrous dimethylformamide at 110 °C (b_1_) to afford 5-substituted tetrazole (Amanpour et al., 2012; Bond et al., 2012). Alternatively, the hydrothermal synthesis was carried out for the synthesis of 5-substituted tetrazole by reacting sodium azide with nitrile using NH_4_Cl, NH_4_F, and propane-1,2-diol (DPOL) for 48 h (b_2_) (Wang et al., 2013). The reaction of the azide ion with nitrile is the most practical way to obtain substituted tetrazole (c) (Wittenberger, 1994). Hydrazoic acid (HN_3_) and organic cyanides (d) were reacted to synthesize 5-substituted tetrazole for the first time through a 1,3-dipolar cycloaddition reaction (Mihina and HERBST, 1950; Vishwakarma et al., 2022).

#### 1.3.3 From amides

**FIGURE 6:**
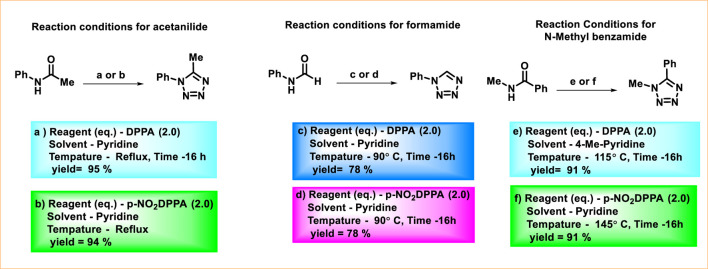
Synthesis of tetrazole from amide derivatives.

Figure 6 illustrates the synthesis of tetrazoles from acetanilide, formamide, and *N-*methyl benzamide. Tetrazoles were synthesized from acetanilide using DPPA or *p*-NO_2_DPPA in reflux conditions with pyridine (a, b). When the reaction was carried out at 90 °C with formamide, high yields of tetrazoles were achieved using either *p*-NO_2_DPPA with pyridine (d) or DPPA with pyridine (c) as the base. On the other hand, the use of *p*-NO_2_DPPA with pyridine (f) afforded high yields of tetrazoles from *N*-methyl benzamide, which contains a bulky substituent adjacent to the carbonyl group. Employing DPPA with 4-methylpyridine as the base (e) provided a comparable yield (Ishihara et al., 2020; Ishihara et al., 2022).

#### 1.3.4 Alcohol and aldehydes

**FIGURE 7:**
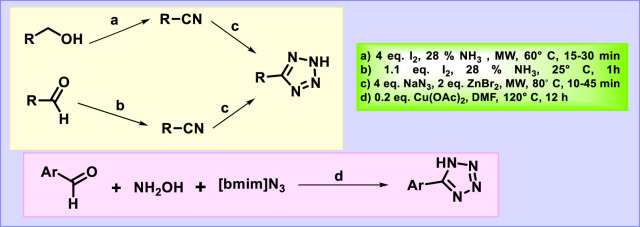
Synthesis of tetrazole from alcohol and aldehydes.

Primary alcohols and aldehydes were treated with iodine in aqueous ammonia under microwave irradiation to produce intermediate nitriles, followed by the [2 + 3] cycloaddition reaction in the presence of sodium azide to synthesize tetrazoles in high yields under microwave conditions (Shie and Fang, 2007). A simple and effective one-pot, three-component reaction was carried out involving aldehydes, hydroxylamine, and [bmim]N_3_ for the synthesis of the 5-substituted 1*H*-tetrazole derivative in Figure 7 (Heravi et al., 2012).

#### 1.3.5 From aryldiazonium salts

**FIGURE 8:**
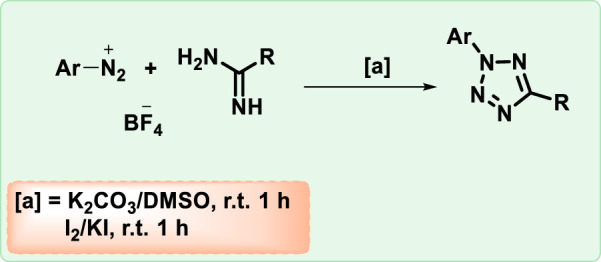
Synthesis of tetrazole from aryldiazonium salts.

A highly efficient one-pot sequential approach has been developed for the direct synthesis of 2,5-disubstituted tetrazoles from aryldiazonium salts and amidines in Figure 8. This method is notable for its short reaction time, mild reaction conditions, and suitability for Gram-scale synthesis (Ramanathan et al., 2015).

### 1.4 Correlation of anticancer and antimicrobial activity

**FIGURE 9:**
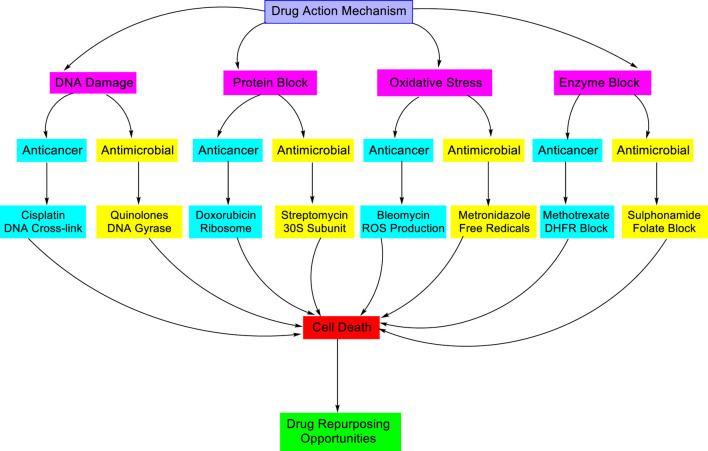
Illustrating the shared and distinct mechanisms of action for anticancer and antimicrobial agents: highlighting overlapping pathways such as DNA damage, protein synthesis inhibition, oxidative stress, and unique cellular targets relevant to therapeutic drug design.

Associating antimicrobial activity with anticancer activity is an area of growing interest in drug discovery and pharmaceutical research. The modes of action, molecular targets, and structural properties of antimicrobial and anticancer agents are quite similar (Figure 9). The anticancer and antimicrobial activities target distinct pathological conditions. Frequently, they have comparable mechanisms of action at the molecular and cellular levels (Martelli and Giacomini, 2018).

#### 1.4.1 Mechanism of action

##### 1.4.1.1 DNA damage or inhibition of DNA replication

The key strategy in both anticancer and antimicrobial therapies is the mechanism of DNA damage or inhibition of DNA replication. These substances work directly by disrupting the replication or damaging the DNA, which eventually kills cancer cells or microbes (Puigvert et al., 2016). Anticancer agents exhibit anticancer activity by targeting the rapid DNA replication in cancer cells. Alkylating agents such as cyclophosphamide and cisplatin produce DNA crosslinks, followed by the prevention of replication. Double-strand breaks are caused by topoisomerase inhibitors like etoposide and doxorubicin. DNA replication is disrupted by antimetabolites such as 5-FU and methotrexate, which mimic or hinder nucleotide synthesis (Swift and Golsteyn, 2014). These actions make them selectively hazardous to rapidly dividing tumor cells at DNA damage checkpoints and trigger apoptosis.

Antibiotics have antimicrobial activity that targets microbial DNA replication to hinder growth. Quinolones/fluoroquinolones such as ciprofloxacin are responsible for DNA breaks by inhibiting DNA gyrase/topoisomerase IV. In anaerobic environments, nitroimidazoles like metronidazole develop reactive species that damage DNA. Rifamycins such as rifampicin inhibit RNA polymerase, and sulfonamides and trimethoprim inhibit folate synthesis, resulting in nucleotide deficiency. These actions disrupt replication and transcription, resulting in bacteriostatic or bactericidal effects by exploiting rapid microbial cell division.

These mechanisms operate effectively on cancer cells and selectively target microbes that replicate faster than normal host cells. DNA damage and other irreversible damage lead to irreparable stress, which ultimately results in cell death (Roos et al., 2016). Repair inhibitors, such as PARP inhibitors, combine with DNA-damaging drugs to potentially enhance their effectiveness (Lodovichi et al., 2020). Tetrazole derivatives can bind to DNA or hinder the replication-related enzymes involved in replication. Structural features such as planar aromatic systems and hydrogen-bond donors enhance DNA binding. Future drug design and development can focus on creating dual inhibitors of cancer and bacterial topoisomerases (Bhattacharya and Chaudhuri, 2008).

##### 1.4.1.2 Protein synthesis inhibition

The inhibition of protein synthesis is a vital mechanism in both anticancer and antimicrobial drug action. Protein synthesis is essential for cell survival, growth, and proliferation. Therefore, agents targeting this mechanism can selectively target cancer cells or other rapidly proliferating pathogens. Cancer cells need high protein synthesis to grow rapidly and exhibit anticancer activity. Rapamycin or other mTOR inhibitors prevent the synthesis of ribosomal proteins (Showkat et al., 2014). Translation initiation inhibitors such as silvestrol disrupt oncogene translation. Homoharringtonine and other elongation inhibitors effectively prevent protein elongation in the treatment of leukemia (Lindqvist and Pelletier, 2009). Proteasome inhibitors primarily inhibit protein degradation, which leads to ER stress and apoptosis (Fribley and Wang, 2006). These mechanisms cause apoptosis, suppress oncogene expression, and inhibit tumor development.

Bacterial ribosomes are different from human ones, which allows for the targeted inhibition of microbial protein synthesis in antimicrobial activity. Tetracyclines prevent the tRNA from binding to the 30S subunit, whereas aminoglycosides such as streptomycin misinterpret the mRNA (Chukwudi, 2016). Macrolides and chloramphenicol act on the 50S subunit, which hampers the peptide elongation or the development of a new bond. Initiation complex formation was inhibited by oxazolidinones, including linezolid. These bacteriostatic or bactericidal activities suppress protein synthesis. Disrupting translation causes stress and cell death because both microbes and cancer cells depend on a significant amount of protein synthesis. Researchers are developing tetrazole derivatives as dual-action inhibitors, which could potentially benefit cancer patients who also experience infection.

##### 1.4.1.3 Oxidative stress generation

Many antibacterial and anticancer drugs with a tetrazole moiety rely significantly on oxidative stress in their mechanism of action. These compounds can make reactive oxygen species (ROS), killing both cancer and microbial cells by damaging their cells. Anticancer agents exploit this phenomenon by increasing ROS levels beyond the cell’s antioxidant defense capacity, resulting in mitochondrial dysfunction, DNA damage, and the initiation of apoptosis or necrosis. Drugs like doxorubicin, arsenic trioxide, and cisplatin cause cell death by generating ROS directly or indirectly. Natural substances like curcumin and resveratrol also increase ROS, especially targeting cancer stem cells (Mirzaei et al., 2023). Redox imbalance, cell toxicity, and apoptotic activation all contribute to the limitation of tumor development. Additionally, ROS-induced signaling may activate caspase cascades, MAPKs, and p53, which could lead to cell cycle arrest or death (Kovacic and Osuna, 2000).

Several antimicrobial agents kill bacteria by triggering oxidative stress through the production of too many ROS. Bacteria have poor antioxidant defenses. Aminoglycosides like gentamicin induce protein misfolding and ROS accumulation (Han et al., 2024), fluoroquinolones such as ciprofloxacin disrupt DNA (Fief et al., 2019), and nitroimidazoles like metronidazole release ROS under anaerobic environments. Silver nanoparticles and other metal-based agents also encourage the ROS formation (Chawla et al., 2023; Sánchez-López et al., 2020). This process leads to damage to bacterial DNA, proteins, and membranes, resulting in potent bactericidal effects that are particularly effective against resistant strains (Albesa et al., 2004; Mourenza et al., 2020; Varshneya et al., 2023).

The broad-spectrum and multitarget characteristics of ROS-based strategies are effective. These strategies can also overcome the problem of resistance. To increase the effectiveness of the drug, researchers can incorporate ROS-generating scaffolds and combine them with antioxidant defense inhibitors. Particularly for patients with impaired immune systems, dual-acting drugs that simultaneously target both cancer and infections hold significant promise.

#### 1.4.2 Shared drug targets or pathways

##### 1.4.2.1 Topoisomerase inhibitors

Topoisomerases are crucial enzymes that regulate the topological state of DNA by reducing the torsional strain generated during replication and transcription (Baranello et al., 2025). Inhibition of these enzymes disrupts critical DNA processes, which causes cell death in both bacteria and cancer cells. In the case of anticancer activity, topoisomerase inhibitors block enzymes essential for DNA replication. Topoisomerase I inhibitors (e.g., topotecan and irinotecan) stabilize single-strand breaks, which turn into lethal double-strand breaks during replication. Topoisomerase II inhibitors (e.g., doxorubicin and etoposide) trap the enzyme-DNA complex after double-strand cleavage, causing persistent DNA damage. These drugs respond against various solid tumors and blood cancers as they trigger apoptosis, especially in rapidly proliferating cancer cells (Liang et al., 2019; Yakkala et al., 2023). Bactericidal agents block the bacterial DNA gyrases topoisomerase II and topoisomerase IV, which inhibit DNA replication, followed by lethal double-strand breakdown for antimicrobial activity (D’Atanasio et al., 2020; Collins and Osheroff, 2024). The interruption of replication and transcription causes bacterial cell death. They are efficacious against a wide range of bacteria, but resistance may develop through gene mutations or efflux mechanisms (Ehmann and Lahiri, 2014). Topoisomerase inhibitors represent a common therapeutic strategy for both microbial and cancer treatment. However, their targets are different for bacterial enzymes in infections and human enzymes in malignant cells. This selectivity facilitated efficient treatment while minimizing toxicity to the host (Pommier et al., 2010).

##### 1.4.2.2 Metabolic pathway inhibitors

Rapidly dividing cells, including bacteria and cancer cells, have a high demand for folate, which is essential for the synthesis of purines and thymidylate, which are the key components of DNA. In case of antimicrobial activity, sulfonamides inhibit dihydropteroate synthase, an enzyme unique to bacterial folate synthesis. Trimethoprim inhibits bacterial dihydrofolate reductase (DHFR), which prevents the transformation of dihydrofolate to tetrahydrofolate (Chawla et al., 2021; Tjampakasari, 2024). This dual blockade inhibits or kills bacteria by interfering with folate metabolism and impairs DNA synthesis. Methotrexate inhibits dihydrofolate from being reduced to tetrahydrofolate by selectively inhibiting the human DHFR, as per its anticancer activity. This procedure successfully interrupts tumor cell division by diminishing the crucial nucleotide precursors for DNA and RNA synthesis (Elgemeie and Mohamed-Ezzat, 2022). Sulfonamides/trimethoprim and methotrexate both target the folate-dependent pathways but inhibit specific enzymes in microbes or human cancer cells. Both drugs are effective against infection and malignancies by interfering with nucleotide synthesis and blocking DNA replication, respectively.

#### 1.4.3 Structural similarities and repurposing potential

Many antimicrobial drugs have a similar type of structural features or mechanisms of action that are also efficient toward cancerous cells due to their common vulnerabilities (Karamanolis et al., 2024). This resemblance has led to significant efforts at cancer therapeutic repurposing. Numerous antimicrobial drugs possess characteristics that increase their anticancer potential, such as lipophilic or cationic structures that damage the cellular membranes and metal-chelating groups that encourage the production of reactive oxygen species (ROS), which are responsible for oxidative damage (Malik et al., 2018).

Many antimicrobial drugs exhibit promising anticancer potential through various mechanisms. Salinomycin targets cancer stem cells (CSCs) by impairing mitochondrial activity and triggering ROS-mediated apoptosis (Qi et al., 2022). Clioquinol interrupts the proteasomal and NF-κB pathways by acting as a metal chelator (Yu et al., 2010). An antidiabetic with antimicrobial properties, metformin blocks mitochondrial complex I, stimulates AMPK, and suppresses mTOR, reducing cancer cell growth (Amengual-Cladera et al., 2024). Azoles such as ketoconazole are beneficial for hormone-dependent cancers by blocking steroid synthesis and drug efflux (Pejčić et al., 2023). Actinomycin D suppresses RNA synthesis for the treatment of pediatric cancers. Cisplatin and doxorubicin exhibit antibacterial efficacy by targeting bacterial topoisomerases and nucleic acid synthesis (Kathiravan et al., 2013). Imatinib inhibits the growth of pathogen replication and regulates immune responses. Structural and mechanistic similarities between antimicrobial agents and anticancer drugs support their repurposing as anticancer therapeutics (Pfab et al., 2021).

#### 1.4.4 Disruption of the microbiome and cancer link

Certain microorganisms, such as *Helicobacter pylori,* are directly associated with the development of cancer by causing long-term inflammation, DNA damage, and increased cell proliferation. Eliminating these microbes is an important method of preventing cancer (Tsukamoto et al., 2017). Additionally, the gut microbiome plays a critical role in controlling the immune system and impacting cancer therapy (Shui et al., 2020). A healthy microbiome improves responses to immunotherapy and chemotherapy, while dysbiosis hinders the therapeutic efficacy and increases side effects (Chrysostomou et al., 2023). Therefore, managing the microbiome is a growing strategy in cancer prevention and treatment.

#### 1.4.5 Immune modulation

In addition to treating infection, antibiotics show an effect on immune responses. Altering the gut microbiota and decreasing essential microbial metabolites and broad-spectrum antibiotics may cause immune modulation, followed by immunosuppression (Zhang and Chen, 2019). On the other hand, some antibiotics, such as macrolides and tetracyclines, have immunostimulatory effects. They exhibit anti-inflammatory and immunomodulatory properties by regulating cytokine production and the activity of immune cells, including macrophages, neutrophils, and T-cells (Ruh et al., 2017). Some beneficial bacteria, including *Bifidobacterium* and *Akkermansia muciniphila,* facilitated antitumor immunity and improved immune checkpoint inhibitors (ICIs) (Huang et al., 2023; Li et al., 2022). On the other hand, antibiotics have the potential to alter and disrupt the microbiome, which reduces the treatment efficacy and patient survival. Integrating microbiome-aware strategies is crucial for optimizing cancer therapy responses because both antibiotics and anticancer drugs showed an impact on the immune function and the microbiome. Antimicrobial and anticancer actions are interconnected through common mechanisms such as oxidative stress induction, metabolic inhibition, and DNA interference. This overlap makes it possible to build dual-activity drugs and comprehend the role of infectious agents in cancer development. Exploring these interconnections may accelerate the discovery of novel therapeutic agents.

Heterocyclic compounds are a valuable resource for discovering and developing new drugs for various medical conditions. More than 85% of chemical compounds with physiological action are heterocycles (Pal et al., 2024). Recently, the tetrazole scaffold has been used to produce promising antimicrobial and anticancer agents. Few review articles have reported on the medicinal importance of tetrazole derivatives as antimicrobial and anticancer agents. Verma et al. (2022) reported synthetic approaches and biological activity of tetrazole analogs against different targets of cancer. Yuan et al. (2024) discussed the potential of tetrazole derivatives with their biological activities against several diseases, such as cancer, bacterial, viral, and fungal infections, asthma, hypertension, Alzheimer’s disease, malaria, tuberculosis, etc. In 2024, Jaiswal and group reported tetrazole derivatives and evaluated biological activities and structure–activity relationships against various neurological disorders, namely, Alzheimer’s, convulsion, depression, Parkinson’s, and diabetic neuropathy (Jaiswal et al., 2024). Atif and Ait Sir (2025) reported the different synthetic approaches of the tetrazole scaffold. These reviews cover either moiety-based aspects or synthetic-based approaches of the tetrazole scaffold. Recent synthetic approaches of tetrazole derivatives with deep insights into SAR for anticancer and antimicrobial activity were not elaborated and discussed in this recent review.

In the current updated review (2023–2025), we discuss recent advancements in synthetic approaches utilizing their core tetrazole moiety to develop compounds that exhibit significant antimicrobial and anticancer activities. The review emphasizes the perspective of structure and spectroscopic properties, physical and chemical properties, the synthetic method of tetrazole formation, the correlation between the mechanism of anticancer and antimicrobial activity, biological activity, and structure–activity relationship of tetrazole derivatives as anticancer and antimicrobial agents.

## 2 Antimicrobial derivatives

**SCHEME 1:**
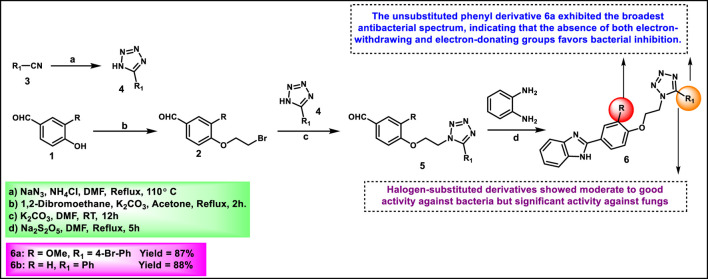
Synthesis and structure–activity relationship studies of benzimidazole-linked tetrazole derivatives as antimicrobial agents.

Rajeswari and coworkers designed and synthesized benzimidazole-linked tetrazole derivatives as antimicrobial agents. They substituted benzaldehyde **1** with 1,2-dibromoethane under basic conditions to produce compound **2**. Substituted benzonitriles **3** were refluxed with sodium azide and ammonium chloride in DMF at 110 °C for 12 h to afford 5-substituted-1*H*-tetrazoles **4** via [3 + 2] cycloaddition. Intermediate **2** was reacted with substituted tetrazole derivatives **4** to yield intermediate **5**. The key aldehyde intermediate **5** was condensed with *o*-phenylenediamine in the presence of sodium metabisulfite in DMF at 110 °C for 5 h. These conditions allowed the intramolecular cyclization to yield the anticipated benzimidazole-tetrazole derivatives **6** (Scheme 1). All the synthesized compounds were tested for antibacterial activity against Gram-positive (*Bacillus subtilis*, *Bacillus megaterium*) and Gram-negative (*Escherichia coli* and *Klebsiella pneumoniae*) strains, and the findings revealed that compound **6b** showed superior activity. Additionally, the researchers conducted antifungal activity tests, which revealed that compounds **6a** and **6b** exhibited significant activity. Antioxidant activity was determined via DPPH and nitric oxide (NO) radical scavenging assays. Compound **6a** showed the most potent radical scavenging activity (IC_50_ = 54.1 μg/mL for DPPH; 75.75 μg/mL for NO) (Rajeswari et al., 2025).

**SCHEME 2:**
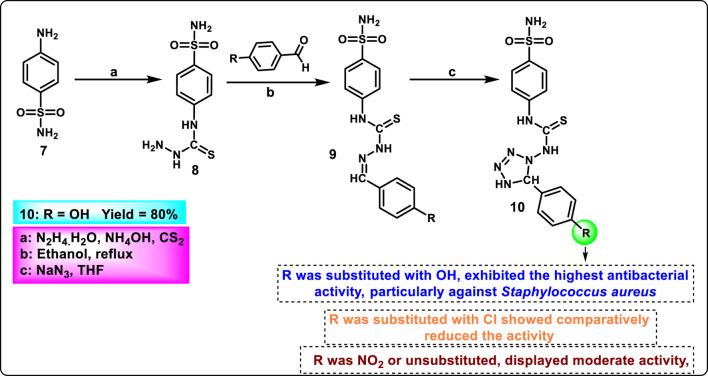
Synthesis and structure–activity relationships study of tetrazole derivatives from sulfonamide.

Saleh et al. designed and synthesized tetrazole derivatives with antibacterial and antifungal activity. Sulfanilamide derivative **7** was treated with hydrazine hydrate and carbon disulfide in the presence of ammonium hydroxide to synthesize intermediate thiosemicarbazide derivative **8**. Then, it was reacted with substituted benzaldehyde to produce a hydrazone derivative via a condensation reaction. In the final step, intermediate **9** underwent a [3 + 2] cyclocondensation reaction in the presence of sodium azide to acquire tetrazole derivative **10** (Scheme 2). The findings of an antibacterial study demonstrated that compound **10** showed the highest activity against *Staphylococcus aureus*, with inhibition zones measuring 30 mm, 25 mm, and 18 mm at concentrations of 0.01 mg/mL, 0.001 mg/mL, and 0.0001 mg/mL, respectively. For *E. coli*, compound **10** exhibited moderate inhibition with a zone of inhibition of 10 mm across all tested concentrations. Antifungal activity was tested against *Candida albicans*, and compound **10** demonstrated significant activity with inhibition zones of 15 mm, 10 mm, and 5 mm at 0.01 mg/mL, 0.001 mg/mL, and 0.0001 mg/mL concentrations, respectively (Saleh et al., 2025).

**SCHEME 3:**
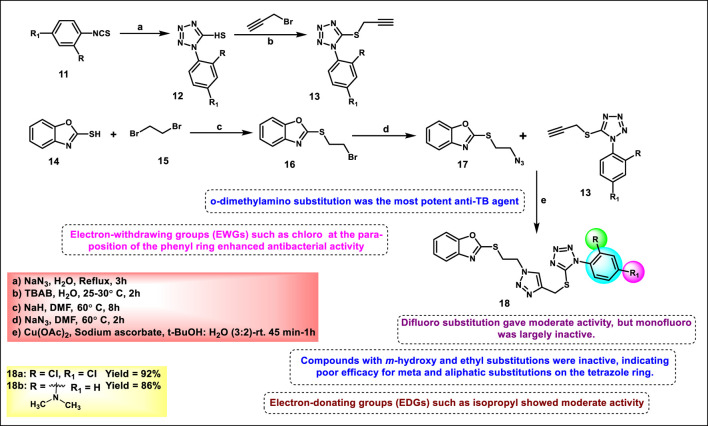
Synthesis of 1,2,3-triazole-incorporated tetrazoles as potent antitubercular agents.

Narkedimilli and coworkers designed and synthesized 1,2,3-triazole-incorporated tetrazoles as potent antitubercular agents. Substituted isothiocyanate **11** was reacted with sodium azide in aqueous conditions to yield substituted tetrazole derivatives **12**, followed by reaction with propargyl bromide in the presence of tetrabutylammonium bromide to yield key intermediate **13** under an aqueous medium. Benzo [*d*]oxazole-2-thiol **14** was reacted with 1,2-dibromoethane **15** in the presence of sodium hydride in dry DMF under ice-cold conditions to yield 2-((2-bromoethyl)thio)benzo [*d*]oxazole **16**, followed by nucleophilic substitution with sodium azide in DMF at 60 °C to obtain 2-((2-azidoethyl)thio)benzo [*d*]oxazole **17**. In the final step, the click reaction was employed to couple the azide **17** and the appropriate alkyne-containing tetrazoles **13** to the synthesis of target compound **18** (Scheme 3). The reaction was carried out in a mixture of tert-butanol and water using copper (II) acetate and sodium ascorbate as the catalytic system at room temperature. Among all the synthesized compounds, compound **18a** exhibited significant broad-spectrum antibacterial activity with minimum inhibitory concentration (MIC) values of 3.8 μg/mL, 5.1 μg/mL, and 3.0 μg/mL against *Pseudomonas aeruginosa*, *Acinetobacter baumannii*, and *Staphylococcus epidermidis*, respectively. Conversely, compound **18b** emerged as the most effective antitubercular agent, exhibiting an MIC of 3.0 μg/mL, which was superior to both streptomycin (5.0 μg/mL) and rifampin (3.9 μg/mL) against the H37Rv strain of *Mycobacterium tuberculosis*. It also had remarkable antibacterial efficacy against *P. aeruginosa*, *S. aureus*, and *S. pyogenes*, with MIC values of 3.0 μg/mL, 1.6 μg/mL, and 2.9 μg/mL, respectively (Narkedimilli et al., 2025).

**SCHEME 4:**
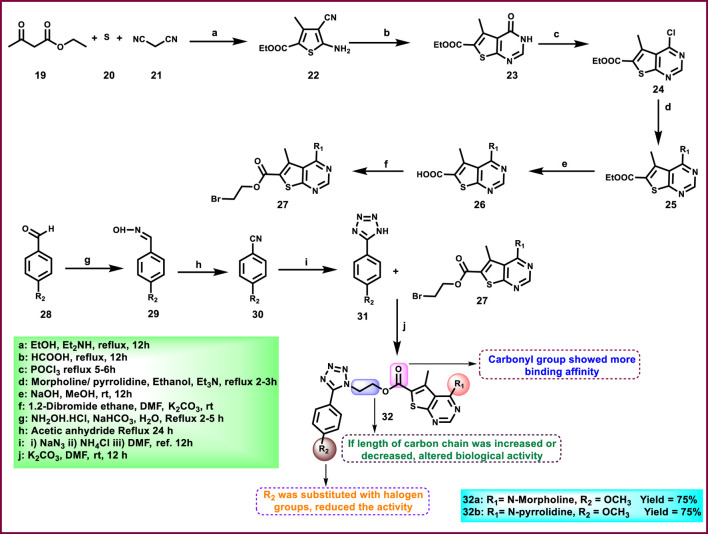
Synthesis of tetrazole-fused pyrimidine derivatives.

Nagaraju and colleagues developed tetrazole-fused pyrimidine derivatives to determine antimicrobial activity. In Scheme 4, ethyl acetoacetate **19**, sulfur **20**, and malononitrile **21** were dissolved in ethanol and triethylamine and refluxed for 12 h to synthesize **22** through Gewald synthesis. Compound **22** underwent cyclization in the presence of formic acid; the chlorination reaction was carried out to obtain **24**. Morpholine and pyrrolidine were added to synthesize **25** from **24** through a nucleophilic aromatic substitution reaction, followed by ester hydrolysis and then esterification to form intermediate **27**. In the next step, 4-methoxybenzaldehyde **28** is converted to 4-methoxybenzaldehyde oxime 29 using NH_2_OH.HCl and NaHCO_3_, followed by a Beckmann rearrangement reaction to yield compound **30**. The mixture of benzonitrile, sodium azide, and ammonium chloride was refluxed at 120 °C to synthesize intermediate **31**. Finally, intermediates **31** and **27** underwent a deprotonation process by nucleophilic substitution reaction to yield final derivatives **32**. The researchers conducted antibacterial activity tests and found that compound **32a** exhibited the highest activity against *S. aureus* and *E. coli*, with inhibition zone diameters of 18.0 mm and 17.70 mm, respectively, at a concentration of 50 μg/mL. Similarly, for *B. subtilis* and*P. vulgaris,* compound **32b** showed the strongest activity with zone inhibition diameters of 16.30 mm and 19.7 mm at a 50 μg/mL concentration. At a 100 μg/mL concentration, compound **32b** showed the most potent activity against *B. subtilis* and *P. vulgaris* with zone inhibition diameters of 18.6 mm and 21.07 mm, respectively. For *S. aureus* and *E. coli,* compound **32a** showed excellent inhibitory activity with zone inhibitory diameters of 19.5 mm and 18.91 mm at a 100 μg/mL concentration. *In vitro* antimicrobial studies indicated that compound **32b** showed the greatest activity, with MIC values of 0.07 μg/mL, 0.09 μg/mL, 0.04 μg/mL, and 0.11 μg/mL against *B. subtilis, E. coli, A. niger,* and *R. oryzae,* respectively, and **32a** showed the highest activity with MIC values of 0.09 μg/mL and 0.13 μg/mL against *E. coli* and *P. vulgaris,* respectively (Nagaraju et al., 2024).

**SCHEME 5:**
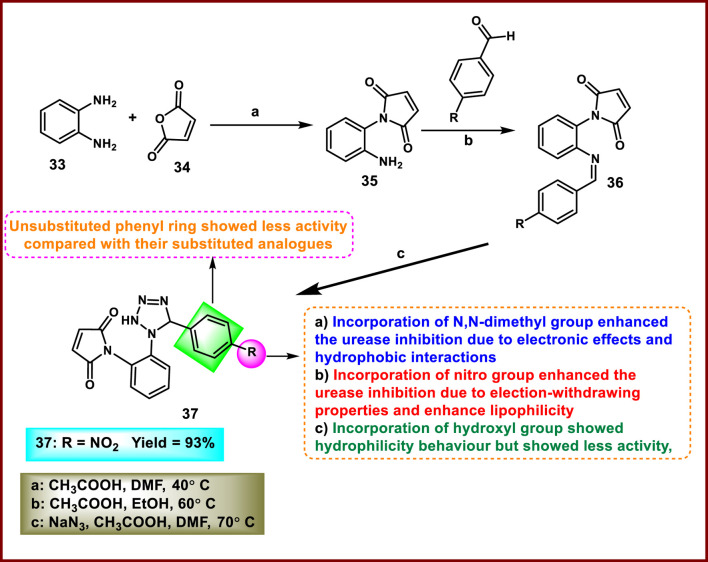
Synthesis of 1-(2-(5-(4-nitrophenyl)-2,5-dihydro-1*H*-tetrazol-1-yl)phenyl)-1*H*-pyrrole-2,5-dione.

Aziz and coworkers designed and synthesized tetrazole-containing pyrrole derivatives as urease inhibitors. Initially, intermediate **35** was obtained by reacting furan-2,5-dione **34** with *o*-phenylenediamine **33** under ultrasonication. Intermediate **35** reacts with substituted benzaldehyde in the presence of acetic acid and ethanol to form Schiff base **36**. Finally, tetrazole derivative **37** was synthesized by reacting sodium azide with Schiff base **36** in DMF via the cycloaddition reaction in Scheme 5. The results of an *in vitro* enzyme inhibitory assay against the urease enzyme revealed that compound **37** showed the highest activity with an IC_50_ value of 4.325 ppm (Muhammed Aziz and Ali Hassan, 2024).

**SCHEME 6:**
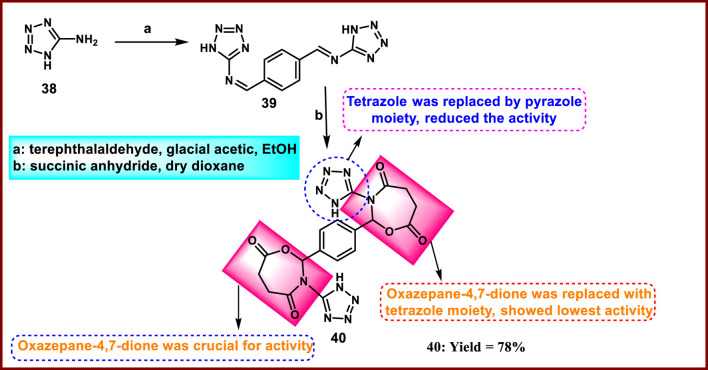
Synthesis and structure–activity relationship of 2,2'-(1,4-phenylene)bis (3-(1*H*-tetrazol-5-yl)-1,3-oxazepane-4,7-dione).

Gailan and the group designed and developed a series of new tetrazole entities as an antimicrobial agent. The Schiff base **39** was prepared via a condensation reaction by mixing 1*H*-tetrazol-5-amine **38** with terephthalaldehyde, followed by the Schiff base undergoing a nucleophilic attack at the carbonyl group of the succinic anhydride to produce the intermediate dipole molecule, which was then transformed into the final derivative **40** (Scheme 6). All the synthesized compounds were evaluated for antibacterial activity, and the results indicated that **40** exhibited the highest antibacterial activity among the tested compounds against *Staphylococcus epidermis*, with a zone of inhibition of 20 mm, while the reference antibiotic tetracycline showed an inhibition diameter of 10 mm at a concentration of 100 mg/mL (Gailan et al., 2024).

**SCHEME 7:**
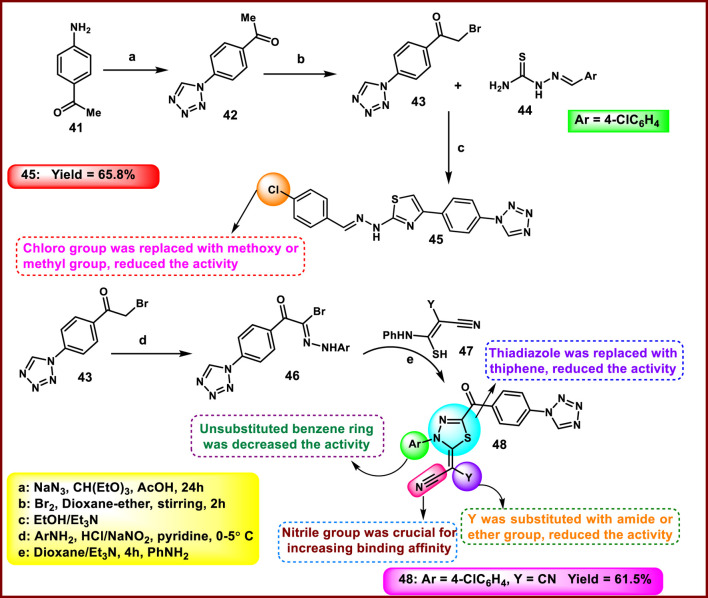
Synthesis and structure–activity relationship of 2-(5-(4-(1*H*-tetrazol-1-yl)benzoyl)-3-substituted-1,3,4-thiadiazol-2(3H)-ylidene)malononitrile derivatives.

Abualnaja et al. synthesized novel tetrazole derivatives and carried out antimicrobial activity. In the first step, 4-aminoacetophenone **41** and sodium azide were refluxed with triethyl orthoformate in an acidic medium to produce the precursor compound 1-(4-acetylphenyl)-1*H*-tetrazole **42,** followed by electrophilic bromination, which afforded intermediate **43**. Intermediate **43** underwent cyclization upon treatment with aromatic thiosemicarbazone **44** to furnish the targeting tetrazole-thiazole hybrids **45** via a Hantzsch-type reaction. Intermediate **43** facilitated the diazo coupling reaction with the diazonium salt obtained to furnish the hydrazonoyl bromide derivative **46** followed by treatment with thiocarbamoyl compound **47** to synthesize target compound **48** in the presence of dioxane and triethylamine (Scheme 7). The antimicrobial activity was tested against *S. aureus, S. pneumoniae, S. typhimurium,* and *E. coli,* and results demonstrated that compound **45** showed the highest activity with MIC values of 6.61 μg/mL and 7.92 μg/mL against Gram-positive bacteria such as *S. aureus* and *S. pneumoniae,* respectively. Meanwhile, **45** showed considerable activity with an MIC value of 28.65 μg/mL against *A. fumigatus*, compared to the reference miconazole MIC value of 25.29 μg/mL; no activity was observed against *C. albicans.* Compound **48** demonstrated the strongest activity against Gram-negative bacteria, *S. typhimurium,* and *E. coli,* with MIC values of 4.27 μg/mL and 5.28 μg/mL, respectively. However, it showed only meager efficacy against *A. fumigatus* and *C. albicans* (Abualnaja et al., 2024).

**SCHEME 8:**
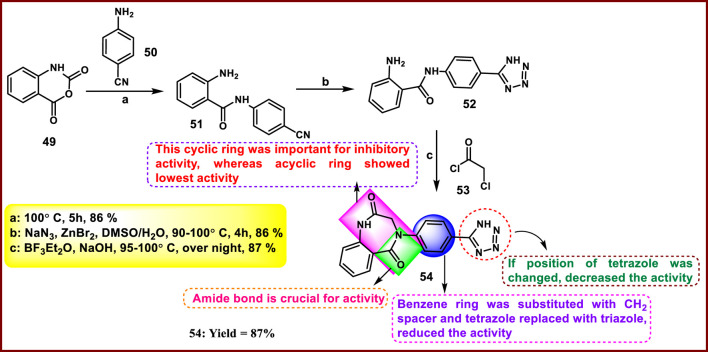
Synthesis of 4-(4-(1*H*-tetrazol-5-yl)phenyl)-3,4-dihydro-1*H*-benzo[*e*][1,4]diazepine-2,5-dione.

Devi et al. discovered tetrazole derivatives as antibacterial agents. Isotonic anhydride **49** reacted with para-amino benzonitriles **50** to synthesize ring-open intermediate **51**, followed by a reaction with sodium azide and zinc bromide to obtain intermediate **52** by a cyclization reaction between nitrile and azide. Then, **52** was treated with 2-chloroacetyl chloride in basic media to obtain the desired compound **54** (Scheme 8). Synthesized compounds were tested for antibacterial activity against *E. coli* and S*. aureus,* and **54** showed the most inhibitory activity with IC_50_ values of 200 μg/mL and 200 μg/mL and MBC values of 390 μg/mL and 340 μg/mL, respectively. In contrast, the reference compound ampicillin exhibited IC_50_ values of 55 μg/mL against *E. coli* and 110 μg/mL against *S. aureus,* respectively (Devi et al., 2023).

**SCHEME 9:**
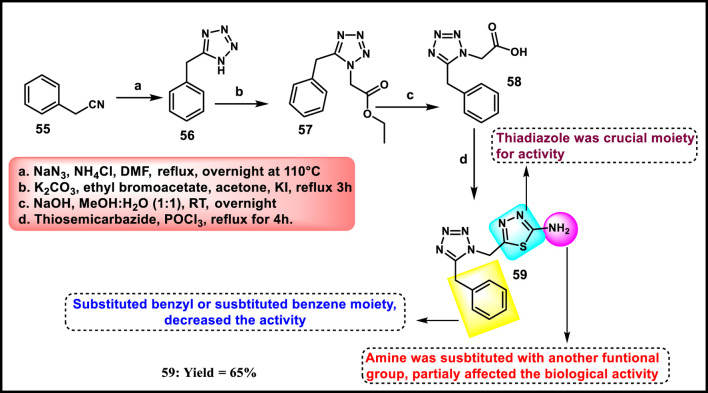
Synthesis and structure–activity relationship of tetrazole-fused thiadiazol derivatives.

Rajeswari et al. reported tetrazole-fused thiadiazole derivatives as antibacterial agents. In Scheme 9, benzyl nitrile **55** and sodium azide were used as starting materials to produce compound **56** through a cyclization reaction, followed by a nucleophilic substitution reaction involving ethyl bromoacetate and potassium iodide, and then ester hydrolysis was performed to obtain intermediate **58**. Finally, an intramolecular cyclization reaction via nucleophilic attack was performed by involving **58** and thiosemicarbazone to produce the tetrazole derivative **59**. All the synthesized compounds were tested for antibacterial activity against *B. subtilis, S. aureus, E. coli,* and *S. aeruginosa*. The results revealed that **59** showed MIC values of 6.25 μg/mL, 1.562 μg/mL, 3.125 μg/mL, and 3.125 μg/mL, respectively. When compared to the standard drug streptomycin, which showed MIC values of 6.25 μg/mL, 3.125 μg/mL, 3.125 μg/mL, and 6.25 μg/mL for the same targets, it appeared that compound **59** exhibited comparable or slightly better antibacterial activity at lower concentrations for some targets (Rajeswari et al., 2024).

**SCHEME 10:**
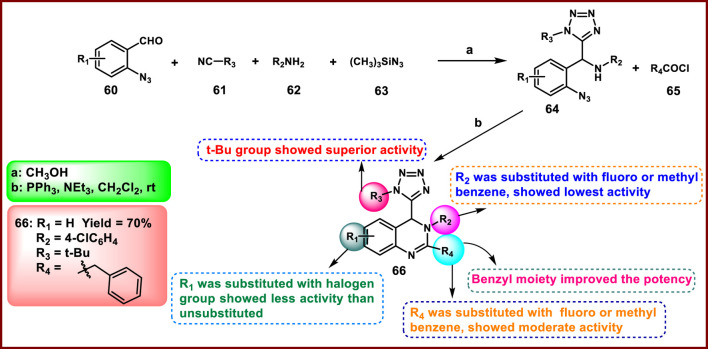
Synthesis and structure–activity relationship of tetrazole-linked quinazoline derivative.

Xu and coworkers developed tetrazole-linked quinazoline derivatives. In Scheme 10, 2-azido-5-chlorobenzaldehyde **60**, t-BuCN **61**, *p*-toluidine **62**, and trimethylsilyl azide **63** were reacted in the presence of methanol to synthesize intermediate **64**, which was further reacted with 2-phenylacetyl chloride **65** in the presence of PPh_3_ under basic conditions to synthesize final derivative **66**. All the synthesized compounds were tested for antibacterial activity against *Microcystis aeruginosa FACHB905,* and results showed that compound **66** showed a growth inhibitory value of 0.063 with 86% inhibition at 100 ppm concentration (Xu et al., 2024).

**SCHEME 11:**
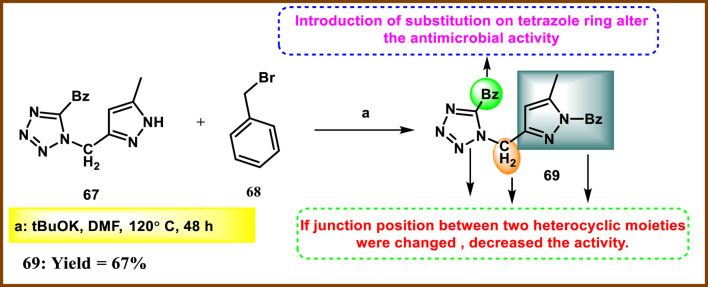
Synthesis of tetrazole-fused pyrazole compounds.

Oulous and coworkers developed tetrazole-fused pyrazole compounds and evaluated antimicrobial activity. Compound **67** and tBuOK were dissolved in DMF and refluxed for 1 h. The process was followed by benzylation to obtain compound **69** (Scheme 11). Six fungal strains, including *Geotrichum candidum*, *Aspergillus niger*, *Penicillium digitatum*, and *Rhodotorula glutinis*, as well as four bacterial strains, *E. coli*, *P. aeruginosa*, *S. aureus*, and *Listeria monocytogenes*, were used to measure the effectiveness of all synthesized compounds. Among these synthesized compounds, **69** showed the highest inhibition potency against bacteria and fungi. For antibacterial activity, **69** showed significant inhibition toward *S. aureus*, *L. monocytogenes*, *E. coli*, and *P. aeruginosa* with inhibition zone diameters of 13 mm, 13 mm, 9 mm, and 9 mm, respectively. Tetracycline, used as a reference compound, showed inhibition potencies of 20 mm, 21 mm, 20 mm, and 22 mm diameter against *E. coli*, *P. aeruginosa*, *S. aureus*, and *L. monocytogenes*, respectively. For antifungal activity, compound **69** exhibited the most potent inhibition against *R. glutinis*, *Saccharomyces*, *G. candidum*, *Aspergillus niger*, and *C. albicans*, with zone inhibition diameters of 18 mm, 11 mm, 14 mm, 12 mm, and 13 mm, respectively. In comparison, the reference compound cycloheximide displayed inhibition diameters of 30 mm, 27 mm, 30 mm, 31 mm, and 35 mm for the same fungi (Oulous et al., 2023).

**SCHEME 12:**
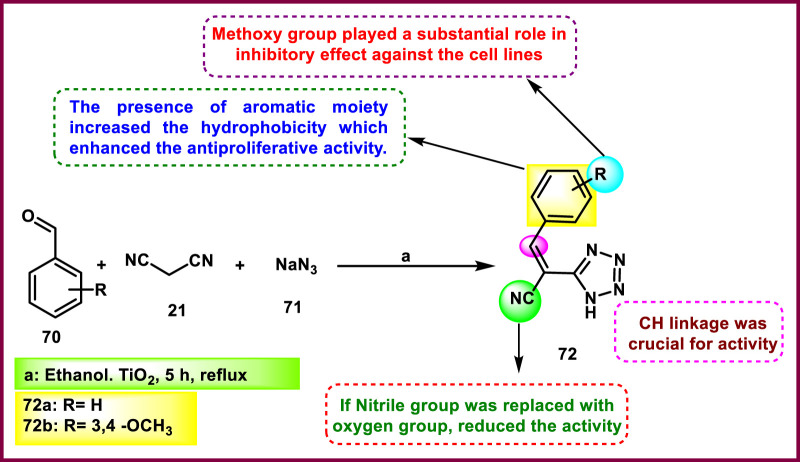
Synthesis and structure–activity relationship studies of 3-(substituted phenyl)-2-(1*H*-tetrazol-5-yl)acrylonitrile.

El-sewedy and coworkers designed and synthesized tetrazole derivatives for dual activities, including antitumor and antimicrobial. In Scheme 12, target derivatives **72a** and **72b** were synthesized using substituted aldehyde **70** with malononitrile **21** and sodium azide **71** in ethanol via aza-Michael addition, followed by a 1,5-endo-dig cyclization reaction in TiO_2_. All the synthesized compounds were subjected to a cytotoxicity study, and results indicated that **72b** showed the most potent antitumor activity against the BJ-1, A431, and HCT116 cell lines with IC_50_ values of 92.045 µM, 44.77 µM, and 201.45 µM, respectively. During antimicrobial screening, **72a** exhibited minimum inhibitory concentration (MIC) values of 6.25 μg/mL and 1.56 μg/mL against *K*. *pneumonia* and *S*. *aureus*, respectively. The MIC value of compound **72b** against *C. albicans* was 12.5 μg/mL. The researchers conducted an antioxidant activity test, which revealed that compound **72b** exhibited the highest radical scavenging activity with percentage inhibitions of 71.7% and 72.5%, while the standard compound ascorbic acid showed inhibitions of 85.4% and 98% at concentrations of 50 mg/mL and 300 mg/mL, respectively, against DPPH. To conduct further research, the lethal dose was calculated for a toxicity study. The results indicated that compound **72b** had a 50% mortality rate with an LD_50_ value of 2500 mg/kg, along with severe symptoms of toxicity, such as weight loss and malaise (El-Sewedy et al., 2023).

**SCHEME 13:**
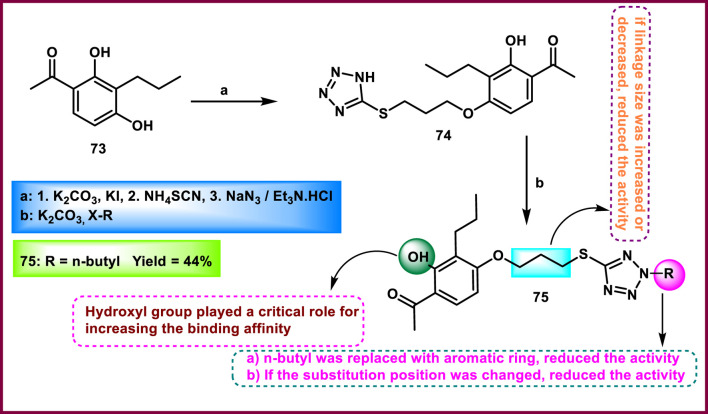
Synthesis of tetrazole-fused hydroxy acetophenone derivatives.

Disli and colleagues developed tetrazole-fused hydroxy acetophenone derivatives and carried out antibacterial activity. 1-(2,4-dihydroxy-3-propylphenyl)ethan-1-one **73** underwent *O*-alkylation, then *S*-alkylation, followed by a cyclization reaction to produce tetrazole intermediate **74** in the presence of potassium carbonate, KI, NH_4_SCN, and NaN_3_/Et_3_N. To yield the target compound **75**, intermediate **74** was subjected to a nucleophilic substitution (SN2) reaction in the presence of an alkyl halide under basic conditions (Scheme 13). Compound **75** exhibited the strongest inhibitory activity against various bacteria and fungi strains, with MIC values of 8 μg/mL against *S. aureus*, 4 μg/mL against *S. epidermidis*, 8 μg/mL against *E. coli,* 16 μg/mL against *P. aeruginosa*, 16 μg/mL against *S. maltophilia*, 16 μg/mL against *C. albicans*, and 4 μg/mL against *A. fumigatus* (Disli et al., 2023).

**SCHEME 14:**
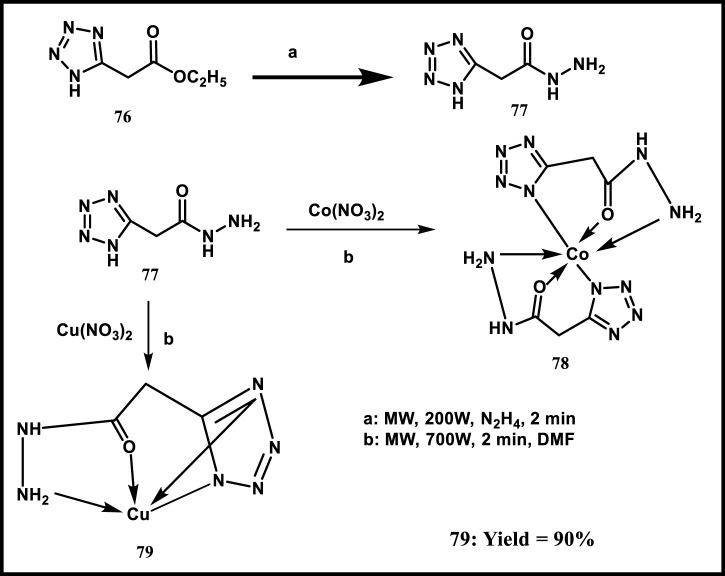
Synthesis of Cu(II) and Co(II) complexes of a synthetic derivative of 2-(1*H*-tetrazol-5-yl)acetohydrazide.

Husain and colleagues developed and synthesized novel Cu(II) and Co(II) complexes of a synthetic derivative of 2-(1*H*-tetrazol-5-yl)acetohydrazide using microwave technology. In Scheme 14, ethyl-1*H*-tetrazole acetate **76** was mixed intimately with hydrazine hydrate. This mixture was heated by MW irradiation for 2 min. The resulting colorless 2-(1*H*-tetrazol-5-yl)acetohydrazide was stirred well in ethanol at 0 °C to obtain ligand **77**. The Co (**78**) and Cu (**79**) complexes of 2-(1*H*-tetrazol-5-yl) acetohydrazide were prepared by mixing the metal nitrate with the ligand using DMF solvent and irradiating for 2 minutes. Regarding the biological activity of spectroscopic studies of DNA interaction, the binding constant (Kb) values with ct-DNA were 0.39 × 10^−3^ M^−1^ for the ligand (**77**), 0.43 × 10^−3^ M^−1^ for the Co complex (**78**), and 0.41 × 10^−3^ M^−1^ for the Cu complex (**79**). The corresponding Ksv values were 0.09 × 10^−1^ M^−1^, 0.11 × 10^−1^ M^−1^, and 0.10 × 10^−1^ M^−1^, respectively. Similarly, the binding constant (Kb) values with BSA for the ligand (**77**), Co complex (**78**), and Cu complex (**79**) were 1.6 × 10^−2^ M^−1^, 3.08 × 10^−2^ M^−1^, and 2.27 × 10^−2^ M^−1^, respectively; the Ksv values were 7.2 × 10^−3^ M^−1^, 98.1 × 10^−2^ M^−1^, and 17.78 × 10^−3^ M^−1^, respectively. Further investigation was carried out using an antioxidant study. The results displayed that the ligand and its complexes demonstrated radical scavenging ability against DPPH, with the order being Co complex (**78**) > Cu complex (**79**) > ligand (**77**) as the concentration increased from 100 μg/mL to 500 μg/mL. This antioxidant activity was significantly lower than that of the well-recognized standard, ascorbic acid. According to antimicrobial investigations, the Co (II) complex **78** outperformed the control antibiotic streptomycin, which could kill opportunistic *S. aureus*, with a zone inhibition diameter of 16.2 mm. Due to their partially planar geometry, Co (II) complexes **78** exhibited the highest activity and potency in all the studies mentioned above. Tetrahedral Cu complexes (**79**) ranked second, followed by the free ligand (**77**) in third place (Husain et al., 2023).

**SCHEME 15:**
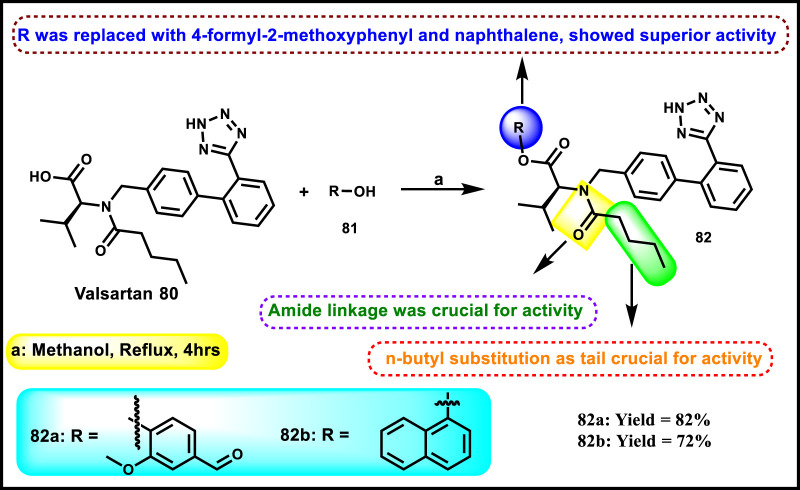
Synthesis of modified valsartan derivatives.

Masood and coworkers reported tetrazole-containing biphenyl derivatives. Valsartan **80** underwent a condensation reaction in the presence of various phenols **81** to obtain target compounds **82** (Scheme 15). All the synthesized compounds were examined for their antihypertensive activity, and compound **82a** demonstrated the greatest percentage of reduction (76.4%) of phenylephrine content on an isolated aorta. The mean systolic blood pressure of 80.17 was measured using the tail-cuff technique after administering compound **82b** (80 mg/kg) to Wistar rats. They also carried out an enzyme inhibition assay on the urease enzyme. The results indicated that compound **82a** displayed the highest inhibitory activity with an IC_50_ value of 0.28 µM, whereas the standard drug thiourea showed an IC_50_ value of 4.24 µM. Additionally, they reported antibacterial activity against *P. aeruginosa*, *S. aureus*, *Bacillus pumilus*, and *E. coli*; unfortunately, none of the synthesized compounds showed antibacterial efficacy (Masood et al., 2023).

**SCHEME 16:**
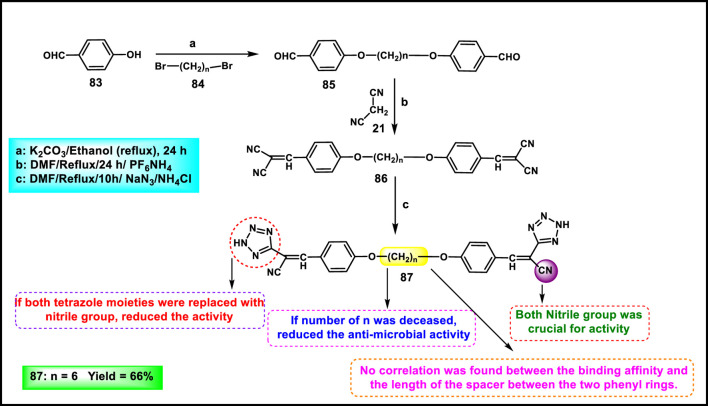
Synthesis of 3,3'-((hexane-1,6-diylbis (oxy))bis (4,1-phenylene))bis (2-(2*H*-tetrazol-5-yl)acrylonitrile).

Bourhou and colleagues synthesized tetrazole derivatives to evaluate their antimicrobial properties. Initially, 4-hydroxybenzaldehyde **83** was refluxed with dibromomethane **84** in ethanol under basic conditions to yield compound **85**. In the second step, these dialdehydes **85** underwent a Michael addition reaction by removal of water and then the formation of a dicyanovinyl group in the presence of malononitrile **21** and ammonium hexafluorophosphate to form **86**. Finally, key intermediate **86** was converted to the corresponding tetrazolic compound **87** via a 1,3-dipolar cycloaddition reaction in the presence of sodium azide in DMF at 110 °C (Scheme 16). The researchers conducted antibacterial activity tests, and the results revealed that compound **87** exhibited the highest activity against *E. coli*, *P. aeruginosa*, *Listeria innocua*, *G. candidum*, *C. albicans*, *R. glutinis*, *A. niger*, and *S. aureus*, with zone inhibition diameters of 14.90 mm, 11.5 mm, 13.45 mm, 12.65 mm, 14.5 mm, 11.25 mm, and 13.65 mm, respectively (Bourhou et al., 2023).

**SCHEME 17:**
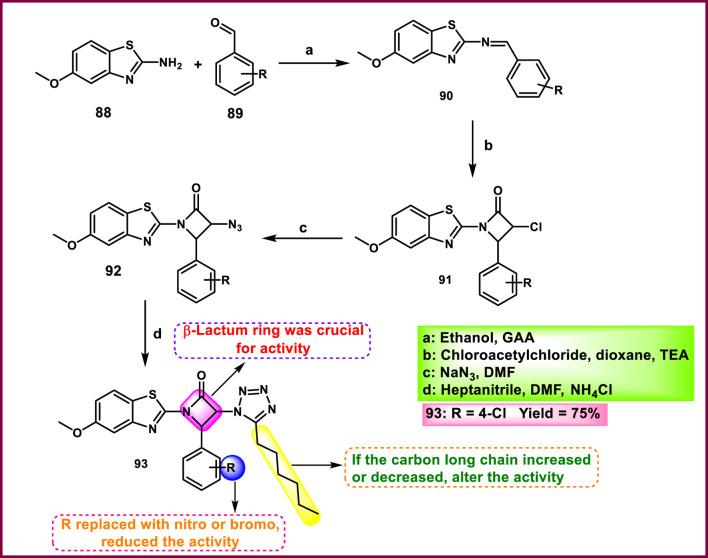
Illustrates the synthesis and structure–activity relationship of 4-(substituted phenyl)-3-(5-hexyl-1*H*-tetrazol-1-yl)-1-(5-methoxybenzo [*d*]thiazol-2-yl)azetidin-2-one.

Ali et al. developed tetrazole derivatives for antimicrobial activity. First, Schiff base derivative **90** was synthesized using amino benzothiazole derivative **88** and substituted benzaldehyde **89** in absolute ethanol under acetic conditions; this was followed by a [2 + 2] cycloaddition or Staudinger reaction, and then a nucleophilic substitution reaction was performed to produce compound **92**. Lastly, targeted tetrazole derivative **93** was synthesized using the azide-nitrile cycloaddition approach from **92** (Scheme 17). They examined antimicrobial activity against bacteria like *E. coli and S. aureus* and fungi such as *C. albicans*. The results indicated that compound **93** exhibited the highest level of activity, as demonstrated by its respective MIC values of 75 μg/mL, 125 μg/mL, and 150 μg/mL. Fluconazole had an MIC value of 250 μg/mL against *C. albicans*, while ampicillin demonstrated MIC values of 100 μg/mL against both *E. coli* and *S. aureu*s(Ali et al., 2023).

**SCHEME 18:**
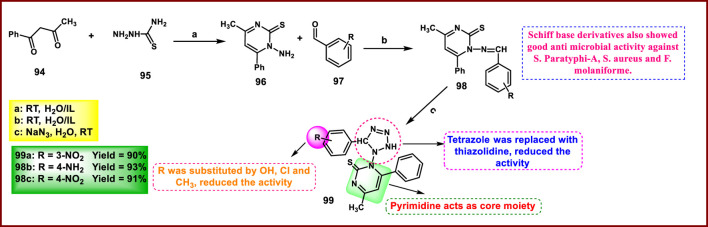
Synthesis of pyrimidine-fused tetrazole derivatives as antimicrobial agents.

Singh and coworkers developed pyrimidine-fused tetrazole derivatives as antimicrobial agents. In Scheme 18, 1-phenylbutane-1,3-dione **94** and hydrazine carbothioamide **95** were reacted in distilled water in the presence of ionic liquid to produce pyrimidine ring derivative **96** through condensation and cyclization, followed by reaction with aromatic aldehyde **97** to yield **98**. Finally, **98** was treated with sodium azide in the presence of distilled water to produce the desired tetrazole-containing complex heterocyclic **99** through the cyclization process. Compound **98b** showed the highest activity with zone inhibition diameters of 17 mm and 19 mm against *S. paratyphi-A* and *S. aureus*, respectively. Compound **99a** showed the highest activity against *E. coli* and *B. subtilis* with zone inhibition diameters of 18 mm and 18 mm, respectively. For antifungal activity, similarly, compound **98c** showed the strongest inhibitory activity against *F. molaniforme*, with a percentage inhibition of 77.22%. Against *A. niger,* compound **99a** showed a percentage inhibition of 69.40%. In contrast, the reference compound Griseofulvin showed percentage inhibition 86% and 81% against *F. molaniforme* and *A. niger* respectively. Other reference compound Fungiguard displayed percentage inhibition 78% and 77% against *F. molaniforme* and *A. niger* respectively (Singh et al., 2023).

**SCHEME 19:**
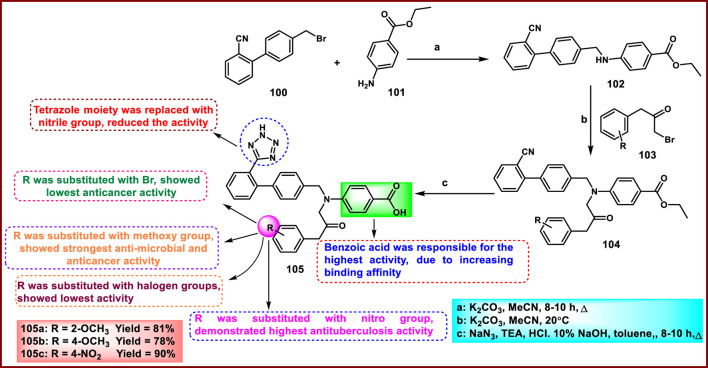
Synthesis of tetrazole-containing biphenyl derivatives through nucleophilic substitution reaction.

Megha et al. developed novel tetrazole-containing biphenyl derivatives and showed dual antimicrobial and anticancer activity. In the first step, a reaction between 4-(bromomethyl)biphenyl-2-carbonitrile **100** and ethyl 4-aminobenzoate **101** underwent a nucleophilic substitution reaction to form the intermediate compound **102**, followed by treatment with substituted 1-bromo-3-phenylpropan-2-one derivatives **103** to generate the compound **104** via nucleophilic substitution reaction (Scheme 19). The cyclization process was carried out to form the tetrazole derivatives **105** in the presence of sodium azide. They exhibited antibacterial activity against *S. aureus*, *B. subtilis, S. typhi,* and *P. aeruginosa.* The results indicated that **105a** exhibited the highest activity with zone of inhibition diameter values of 15.2 mm, 15.0 mm, 15.0 mm, and 15.3 mm, respectively, at higher concentrations (100 μg/mL), which is similar to the standard drug gentamycin. The researchers also conducted a cytotoxicity study, revealing that compound **105b** exhibited the strongest inhibitory action, with IC_50_ values exceeding 8.47 μg/mL against the A549 cell line, which is very close to the standard drug doxorubicin’s IC_50_ value of 7.60 μg/mL. Additionally, they looked into the anti-tuberculosis activity, and the results showed that compound **105c** had high sensitivity with MIC values of 3.12 μg/mL, which was similar to the reference standard pyrazinamide (MIC = 3.12 μg/mL) (Megha and Bodke, 2023).

**SCHEME 20:**
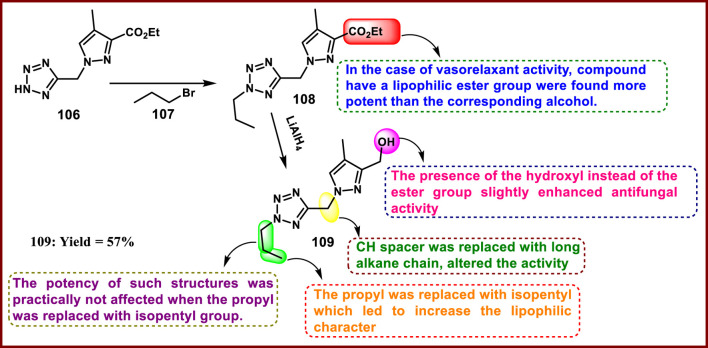
Synthesis and structure–activity relationships of (4-methyl-1-((2-propyl-2*H*-tetrazol-5-yl)methyl)-1*H*-pyrazol-3-yl)methanol.

Cherfi and coworkers developed pyrazole-fused tetrazole derivatives. In Scheme 20, a mixture of **106** and tBuOK in THF was heated under reflux in the presence of 1-bromopropane **107** to obtain intermediate **108** via *N*-alkylation. Intermediate **108** underwent ester reduction in the presence of LiAlH_4_ to synthesize compound **109**. All synthesized compounds were tested for antifungal activity, and results demonstrated that compound **109** showed the highest activity with inhibition diameters of 15 mm, 15 mm, 15 mm, and 14 mm against *G. candidum, A. niger, P. digitatum,* and *R. glutinis,* respectively. Additionally, they investigated the vasorelaxant action on an isolated rat aorta, and the outcomes revealed that compound **108** had the most significant activity with a maximum effect (E_max_) of 60.26%. Carbachol and verapamil were employed in this investigation as positive controls and showed E_max_ values of 85.52% and 91.62%, respectively (Harit et al., 2023).

**SCHEME 21:**
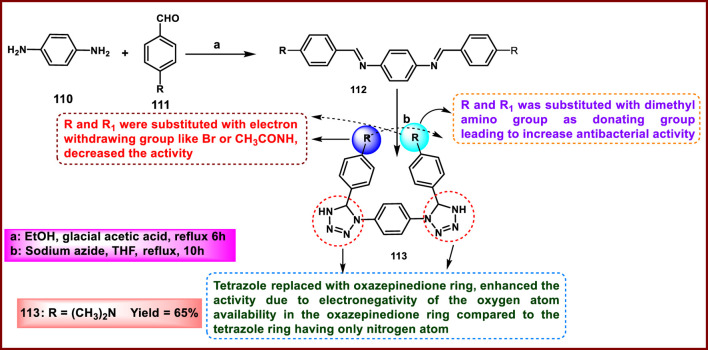
Synthesis and structure–activity relationship studies of phenylene-bis-tetrazole derivatives.

Beebany and coworkers developed new phenylene-bis-tetrazole derivatives and tested them against bacteria. First, intermediate bisimine **112** was synthesized from the reaction between aldehydes **111** and amines **110** using absolute ethanol as a solvent. These imines have been transformed into heterocyclic derivative **113** via the cyclocondensation reaction of imines with phthalic anhydride and sodium azide (Scheme 21). They carried out an antibacterial activity against Gram-negative *E. coli* and Gram-positive *S. aureus* to assess their potential as antibacterial agents. The results showed that compound **113** showed the highest inhibition performance against the growth of the applied bacteria. Furthermore, compound **113** exhibited increased activity at higher concentrations, resulting in a larger inhibition zone. These data suggest that its antibacterial effectiveness is dose-dependent, with higher concentrations leading to enhanced inhibition of bacterial growth. Compound **113** showed MIC values of 31 mm and 21 mm at 0.01 μg/mL concentration and MIC values of 12 mm and 13 mm at 0.0001 μg/mL concentration against *E. coli* and *S. aureus,* respectively (Beebany, 2023).

**SCHEME 22:**
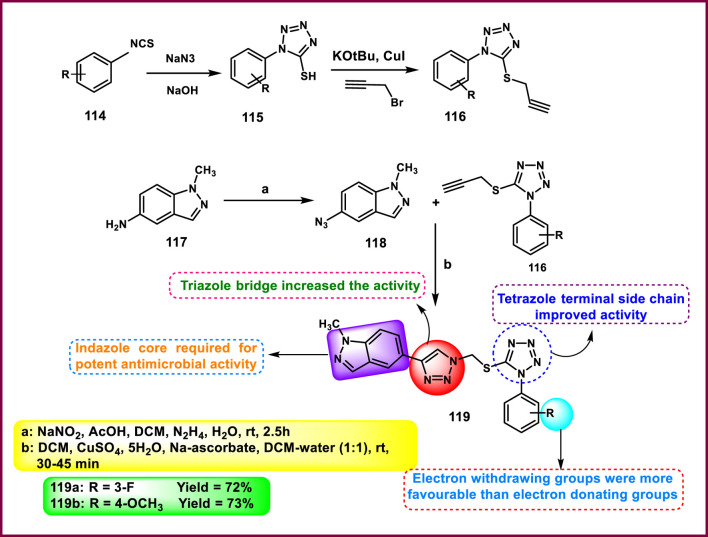
Synthesis of triazolyl tetrazole-bearing indazole derivatives as antimicrobial agents.

Gujja and coworkers focused on the development of triazolyl tetrazole-bearing indazole derivatives as antimicrobial agents. Phenyl isothiocyanate **114** underwent a cyclization reaction in the presence of sodium azide to give 1-phenyl-1*H*-tetrazole-5-thiol **115**, followed by deprotonation and a nucleophilic substitution reaction to produce derivative **116**. In the presence of catalytic amounts of sodium nitrite, 1-methyl-1*H*-indazol-5-amine **117** was converted to compound **118** under a nitrogen atmosphere, which underwent Cu(I)-catalyzed azide-alkyne cycloaddition or click reaction, involving intermediate **116**, to yield compounds **119** in the presence of DMF and copper sulfate under a nitrogen atmosphere (Scheme 22). All the synthesized compounds were tested for antimicrobial activity. The results showed that compound **119a** showed superior inhibitory activity against *S. aureus, B. subtilis, E. coli, P. aeruginosa,* and *A. flavus* with MIC values of 5 μg/mL, 10 μg/mL, 12 μg/mL, 13 μg/mL, and 18 μg/mL, respectively, and compound **119b** demonstrated the highest activity against *M. Luteus* and *M. gypseum* with MIC values of 9 μg/mL and 11 μg/mL, respectively (Gujja et al., 2023).

**SCHEME 23:**
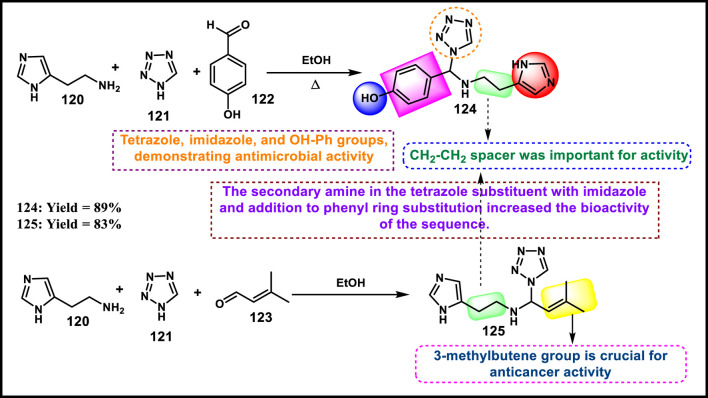
Synthesis of imidazole-containing tetrazole derivatives.

Loganathan et al. designed and synthesized tetrazole derivatives that were screened for dual antimicrobial and anticancer activity. In Scheme 23, a mixture of equivalent moles of 2-(1*H*-imidazole-5-yl)ethanamine **120**, 1*H*-tetrazole **121**, and benzaldehyde **122** or 3-methylbut-2-enal **123** in ethanol with HCl was refluxed for 2 h at 60 °C to form compounds **124** and **125**, respectively, through a condensation reaction. All the synthesized compounds were tested for antibacterial activity *against S. aureus, E.* coli, *E. faecalis, P. aeruginosa,* and *K. pneumoniae*. The results showed that compound **124** showed the highest activity with MIC values of 2 μg/mL, 4 μg/mL, 8 μg/mL, 32 μg/mL, and 8 μg/mL, respectively. Those showed similar activity to the reference compound, cefazolin. For antifungal activity, compound **124** showed the highest inhibitory activity against *A. niger*, *M. audouinii, C. albicans,* and *C. neoformans,* with MIC values of 2 μg/ML, 1 μg/mL, 16 μg/mL, and 32 μg/mL, respectively. To further investigate, the researchers conducted cytotoxicity assays, which revealed that compound **124** exhibited remarkable efficacy against the HeLa cell line with a GI_50_ value of 0.01 µM, while compound **125** demonstrated superior activity against the HepG2 and MCF7 cell lines with GI_50_ values of 0.02 µM and 0.04 µM, respectively. The standard compound doxorubicin showed GI_50_ values of 0.01 µM against the HepG2 cell line, 0.02 µM against the MCF7 cell line, and 0.04 µM against the HeLa cell line, respectively (Loganathan et al., 2024).

**SCHEME 24:**
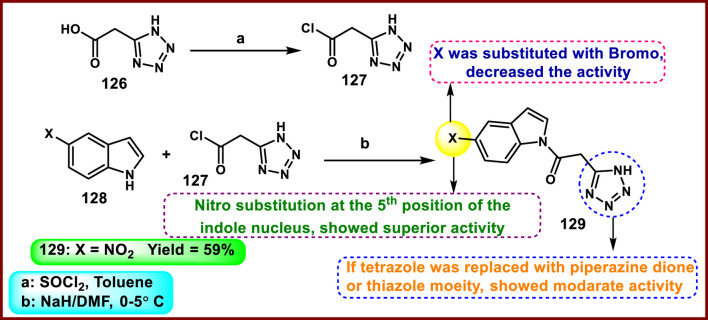
Synthesis and structure–activity relationship studies of *N*-acyl indole-substituted tetrazole derivatives.

Jagadeesan and coworkers developed *N*-acyl indole-substituted tetrazole derivatives that screened for dual antimicrobial and anticancer activity. 2-(1*H*-tetrazol-5-yl)acetic acid **126** underwent a chloroacetylation reaction in the presence of SOCl_2_ to prepare intermediate **127**, followed by a nucleophilic substitution reaction with indole derivative **128** to produce the desired compound **129** (Scheme 24). Synthesized derivatives were tested for *in vitro* antibacterial activity against *S. aureus MTCC 737*, *K. planticola MTCC 2277, B. cereus MTCC 430, S. aureus MLS16 MTCC 2940, E. coli MTCC 1687,* and *P. aeruginosa MTCC 424*. The results revealed that compound **129** showed the highest activity with MIC values of 18.75 μg/mL, 9.37 μg/mL, 37.5 μg/mL, 9.37 μg/mL, 1.67 μg/mL, and 18.75 μg/mL, respectively. They tested antifungal activity, and according to their data, compound **129** showed superior inhibitory activity against *A. niger*, equal to that of the standard miconazole’s MIC value of 9.37 μg/mL. The results of a cytotoxicity study showed that compound **129** demonstrated the highest activity against the HeLa, A549, MCF-7, and K562 cell lines with IC_50_ values of 3.8 µM, 47.5 µM, 2.4 µM, and 6.9 µM, respectively (Jagadeesan and Karpagam, 2023).

**SCHEME 25:**
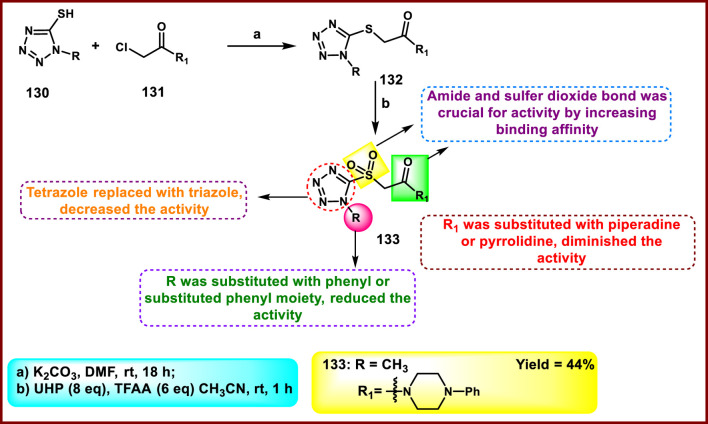
Synthesis and structure–activity relationships of tetrazole-containing sulfonyl acetamides congeners.

Henches and coworkers developed tetrazole-containing sulfonyl acetamide moieties as inhibitors of *M. tuberculosis* and *Mycobacterium marinum*. This synthetic route in Scheme 25 started with the commercially available 5-mercapto-1-substituted tetrazole **130**, which was *S*-alkylated with compound **131** to afford intermediate **132**. The use of UHP and TFAA in acetonitrile resulted in the predominant formation of the desired sulfones (**133**). All the synthesized compounds were tested for *in vitro* inhibitory activity against *M. tuberculosis* and *M. marinum. The results* demonstrated that compound **133** showed the highest activity with MIC_90_ values of 1.25 μg/mL and 10 μg/mL, respectively, while the standard drug rifampin showed superior efficacy with MIC_90_ values of 0.06 μg/mL and 0.2 μg/mL for the same targets (Henches et al., 2023).

**SCHEME 26:**
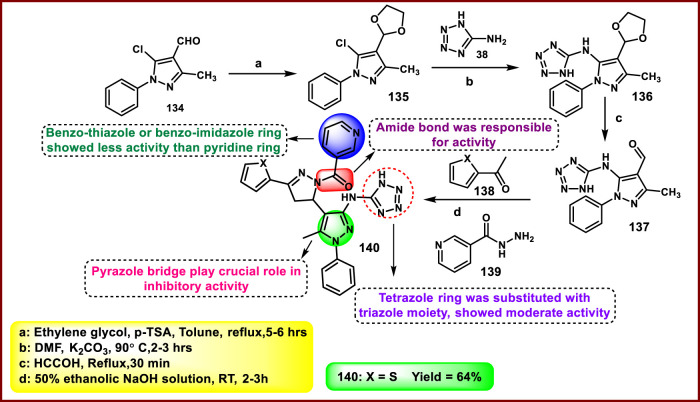
Synthesis of pyrazolyl pyrazoline-clubbed tetrazole hybrids as anti-TB agents.

Zala and coworkers developed pyrazolyl pyrazoline-clubbed tetrazole hybrids as promising anti-tuberculosis agents. In Scheme 26, 5-chloro-4-(1,3-dioxolan-2-yl)-3-methyl-1-phenyl-1*H*-pyrazole **135** was synthesized by reaction with derivative **134** and ethylene glycol using *p*-toluene sulfonic acid (*p*-TSA) in toluene under reflux conditions via acetalization reaction, followed by nucleophilic substitution reaction, then deprotection to yield compound **137**. Finally, **137** was reacted with 2-acetyl furan **138** and hydrazide derivative **139** in an ethanolic NaOH solution to synthesize compound **140** through nucleophilic addition and cyclization reaction. All the synthesized compounds were tested for *in vitro* antitubercular activity against the *M. tuberculosis* H37Rv strain. The results demonstrated that compound **140** showed the highest activity, with MIC values of 12.5 μg/mL and a percentage inhibition of 99% (Zala et al., 2023).

**SCHEME 27:**
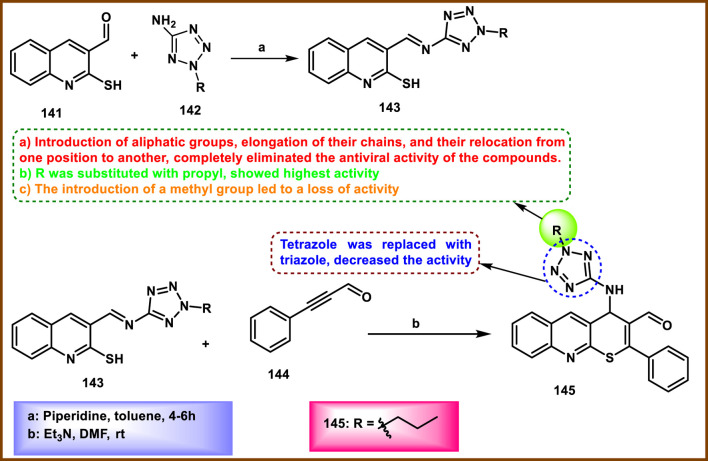
Synthesis of tetrazole-fused quinoline derivatives.

Khramchkhin et al. developed tetrazole-fused quinoline derivatives and evaluated their antiviral activity. In Scheme 27, 2-propyl-2*H*-tetrazol-5-amine **142** has a nucleophilic amine group, which attacks the electrophilic carbonyl carbon of the 2-mercaptoquinolin-3-carbaldehyde **141** to create azomethine derivatives **143**. Compound **143** further reacted with 3-phenylpropiolaldehyde **144** to synthesize the final compound **145** in triethylamine and DMF through conjugate addition (Michael addition) followed by an intramolecular cyclization process. Among these synthesized compounds, **145** showed excellent potency against the influenza A/Puerto Rico/8/34 virus in the MDCK cell line with IC_50_ values of 18.4 µM and a selectivity ratio (SI) value of >38. A drug with a higher SI ratio is likely to be safer and more effective in treating a viral infection. In contrast, the standard drug oseltamivir carboxylate has an IC_50_ value of 0.17 µM and an SI value greater than 588 (Khramchikhin et al., 2023).

## 3 Anticancer derivatives

**SCHEME 28:**
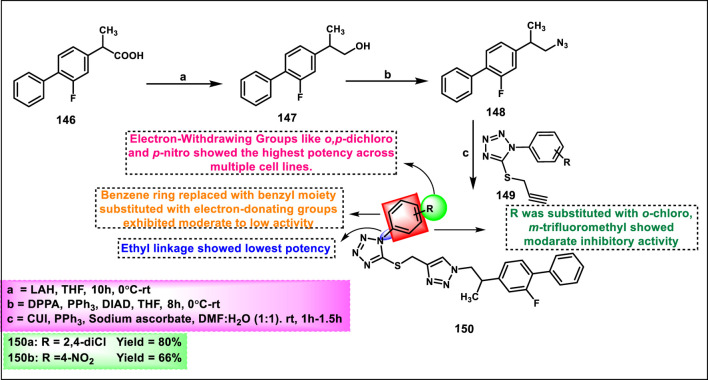
Synthesis of 1,2,3‒triazole-fused tetrazole hybrids as anticancer agents.

Vellaiyan et al. designed and synthesized 1,2,3‒triazole-fused tetrazole hybrids as anticancer agents. The process begins with material 2-(2-fluoro-[1,1′-biphenyl]-4-yl) propanoic acid **146**, followed by a nucleophilic addition reaction using lithium aluminum hydride in THF at room temperature to obtain intermediate **147**. This stage is then followed by a nucleophilic substitution to form alkyl azide **148** by reacting diphenyl phosphoryl azide (DPPA) in the presence of DIAD and PPh_3_. In the final step, Cu(I)-catalyzed cycloaddition was carried out between the terminal alkyne of **149** and the azide of intermediate **148** to form a regioselective 1,4-disubstituted 1,2,3-triazole ring formation and yield target compound triazole-fused tetrazole derivatives **150** (Scheme 28). The antiproliferative assay revealed that compound **150a** exhibited the highest activity against the HepG2, HuH7, and MCF-7 cell lines with IC_50_ values of 1.83 μM, 1.46 μM, and 1.35 μM, respectively, and compound **150b** showed activity against the T-47D cell line with an IC_50_ value of 3.55 μM (Vellaiyan et al., 2025).

**SCHEME 29:**
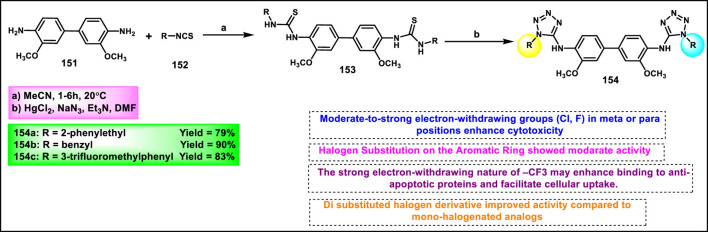
Synthesis and structure–activity relationship studies of tetrazole derivatives.

Olejarz and coworkers designed and developed novel tetrazole derivatives as Bcl-2 apoptosis regulators for the treatment of cancer. Initially, 3,3′-dimethoxybenzidine **151** was treated with various aryl or alkyl isothiocyanates **152** in dry acetonitrile under ambient conditions for 1–6 h, resulting in bis-thiourea intermediates **153**. It was followed by a cyclization reaction with sodium azide to synthesize the final target compound **154** in the presence of mercuric chloride, dry DMF, and triethylamine (Scheme 29). All the synthesized compounds were tested against human cancer cell lines like HTB-140, A549, HeLa, and SW620. The findings revealed that compound **154b** showed the highest activity against respective cell lines with IC_50_ values of 23.5 μM, 34.2 μM, 20.3 μM, and 21.3 μM. Flow cytometric analysis using annexin V/7-AAD staining revealed that **154c** showed the highest late apoptotic activity in A549 cells (92.1% late apoptosis). Molecular docking studies identified compound **154a** as a potent Bcl-2 protein inhibitor (Olejarz et al., 2025).

**SCHEME 30:**
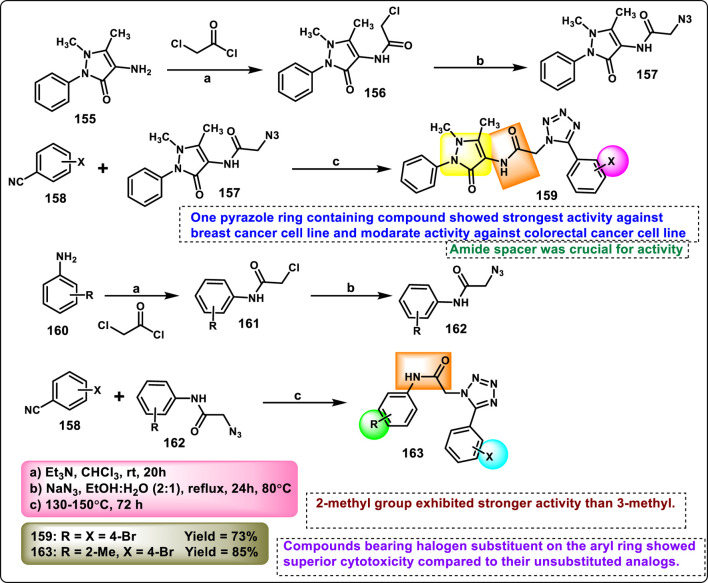
Synthesis of pyrazole clubbed tetrazole derivatives as anticancer agents.

Manwar and coworkers developed novel tetrazole derivatives as anticancer agents. Phenyl pyrazole derivative **155** and aniline derivative **160** were reacted with chloroacetyl chloride to obtain corresponding chloroacetamide derivatives **156** and **161**, which reacted with sodium azide (NaN_3_) in a mixture of ethanol and water to synthesize azide intermediates **157** and **162**. The azide intermediates were treated with 4-bromobenzonitrile **158** in a sealed vial at 130 °C for 72 h under solvent-free conditions to obtain final derivatives. The regioselective [3 + 2] cycloaddition reaction produced 1-substituted-5-aryl tetrazole derivatives **159** and **163** (Scheme 30). All the synthesized compounds were tested for *in vitro* cytotoxicity assays against two human cancer cell lines, HT-29 and MDA-MB-231. The findings showed that compound **163** showed the highest activity against the HT29 cell line, and compound **159** showed superior activity against the MDA-MB-231 cell line with IC_50_ values of 69.99 μg/mL and 86.73 μg/mL, respectively (Manwar et al., 2025).

**SCHEME 31:**
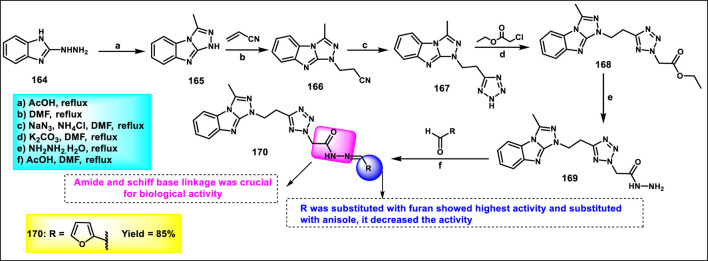
Synthesis of benzimidazole–triazole–tetrazole derivatives as anticancer agents targeting breast cancer.

Rahman and coresearchers developed benzimidazole-triazole-tetrazole derivatives as anticancer agents targeting breast cancer. Hydrazine derivative **164** reacts with acetic acid to form compound intermediate **165** via an intramolecular cyclization reaction. The Michael addition reaction, followed by a [3 + 2] cycloaddition reaction, was carried out to synthesize compound **167** in the presence of sodium azide and ammonium chloride. Thereafter, intermediate **167** underwent an *N*-alkylation reaction via the SN_2_ mechanism in the presence of ethyl chloroacetate under basic conditions to yield ester derivative **168**, followed by hydrazinolysis in the presence of hydrazine hydrate to form hydrazide derivative **169**. In the final step, the hydrazide derivative **169** reacted with furfural to form the target compound, the Schiff base derivative **170** (Scheme 31). An *in vitro* cytotoxicity assay revealed that compound **170** demonstrated superior activity against the MCF-7 cell line with an IC_50_ value of 0.29 μM, whereas 5-fluorouracil showed an IC_50_ value of 0.11 μM. A molecular docking study was also performed, and compound **170** showed the highest binding affinity against the 8GVZ protein with a binding affinity of −6.68 kcal/mol and formed a hydrogen bond with the Thr1782 amino acid residue (Abdel-Rahman et al., 2025).

**SCHEME 32:**
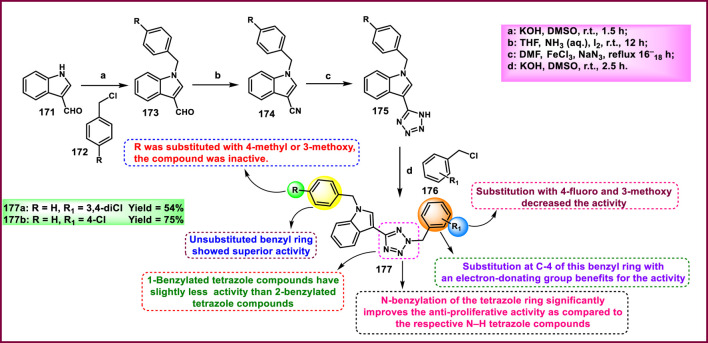
Synthesis and structure–activity relationship of indole-based tetrazole derivatives as anti-breast cancer agents.

Kaur and coworkers developed indole-based tetrazole derivatives as an anti-breast cancer agent. The *N*-position of the initial compound 1*H*-indole-3-carbaldehyde **171** underwent benzylation reactions using benzyl chlorides **172** in basic conditions to yield compound **173**. Furthermore, the aldehyde-to-nitrile transformation reaction was performed using an ammonia-based condensation followed by an oxidation reaction to yield intermediate **174** with iodine. Following this, an azide-nitrile cycloaddition reaction formed a tetrazole derivative **175**. In the final step, *N*-benzylation of indole–tetrazoles **175** in the presence of KOH using substituted benzyl chlorides occurred with excellent yields of 2-benzylated tetrazole derivatives **177** (Scheme 32). All the synthesized compounds were subjected to an antiproliferative assay, and results revealed that compound **177b** showed the highest activity with IC_50_ values of 3.83 µM, 7.83 µM, and 15.7 µM against the T-47D, MCF-7, and MDA-MB-231 cell lines, respectively. Compound **177a** showed the second-highest inhibitory activity with IC_50_ values of 10.00 µM, 7.95 µM, and 19.4 µM against the T-47D, MCF-7, and MDA-MB-231 cell lines, respectively. The most active compounds, **177a** and **177b**, were selected to determine their cytotoxicity against normal human embryonic kidney cells, HEK-293. Compound **177a** was found to have non-significant cytotoxicity against the HEK-293 cells, while compound **177b** has much less cytotoxicity (IC_50_ = 108.6 µM) against the normal cells than the standard drug bazedoxifene (IC_50_ = 38.48 µM). To determine the ER-α binding affinity, compounds **177a** and **177b** were tested for the ER-α competitor assay. Based on the competitive binding experiment findings, compounds **177a** and **177b** showed IC_50_ values of 5.826 nM and 110.6 nM, respectively, demonstrating stronger binding affinity and greater efficiency for ER-α than the standard drug bazedoxifene, which has an IC_50_ of 339.2 nM. For further investigation, they carried out Western blotting, and according to the results, compound **177a** induced 46.06% expression of ER-α protein in T-47D cells. This result suggests that the compound **177a** inhibited the expression of ER-α protein in T-47D cells to exhibit its anticancer activity (Kaur et al., 2024).

**SCHEME 33:**
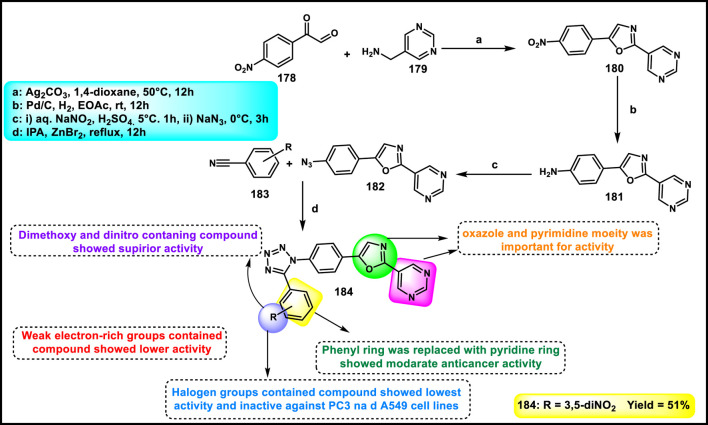
Synthesis of tetrazole ring-incorporated oxazole-pyrimidine derivatives.

Asiri and colleagues designed and developed tetrazole ring-incorporated oxazole-pyrimidine derivatives as anticancer agents. In Scheme 33, 2-(4-nitrophenyl)-2-oxoacetaldehyde **178** was reacted with pyrimidin-5-yl-methanamine **179** in the presence of Ag_2_CO_3_ to produce the pure compound 5-(4-nitrophenyl)-2-(pyrimidin-5-yl)oxazole **180** through a cyclization reaction, followed by reduction to yield amine intermediate **181**. Under the Sandmeyer reaction conditions, intermediate **181** was changed into azide intermediate **182**, followed by a cyclization reaction in the presence of substituted benzonitrile **183** and ZnBr_2_, which produced compound **184** in good yields. *In vitro* cytotoxicity assay revealed that, among these synthesized compounds, compound **184** exhibited the highest inhibitory activity against the PC3, A549, MCF-7, and DU-145 cell lines with IC_50_ values of 0.08 µM, 0.04 µM, 0.01 µM, and 0.12 µM, respectively. The standard compound etoposide showed IC_50_ values of 2.39 µM, 3.08 µM, 2.11 µM, and 1.97 µM, respectively (Asiri et al., 2024).

**SCHEME 34:**
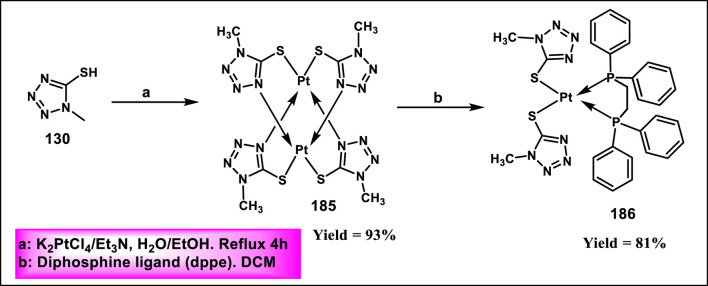
Synthesis of platinum complex containing tetrazole derivatives as promising anticancer agents.

Al-Samrai et al. developed a platinum complex containing tetrazole derivatives as a promising anticancer agent. Complex **185** was prepared by reacting two equivalents of 1-methyl-1*H*-1,2,3,4-tetrazole-5-thiol **130** with one equivalent of K_2_PtCl_4_ in the presence of Et_3_N as a basic medium. Additionally, an equivalent molar amount of complex **185** and diphosphine ligands (diphos = dppe) was reacted in dichloromethane as a solvent, yielding complex **186** (Scheme 34). Researchers evaluated the enzyme inhibition assay against alkaline phosphatase (ALP), and the finding revealed that complex **186** exhibited the inhibitory percentage (84.02%) at a concentration of 10^–4^ mol/L. For additional investigation, they conducted cell line activity against the HepG2 cell line, and findings indicated that complex **185** demonstrated the highest activity, with an IC_50_ value of 34.78 μM (Al-Samrai et al., 2024).

**SCHEME 35:**
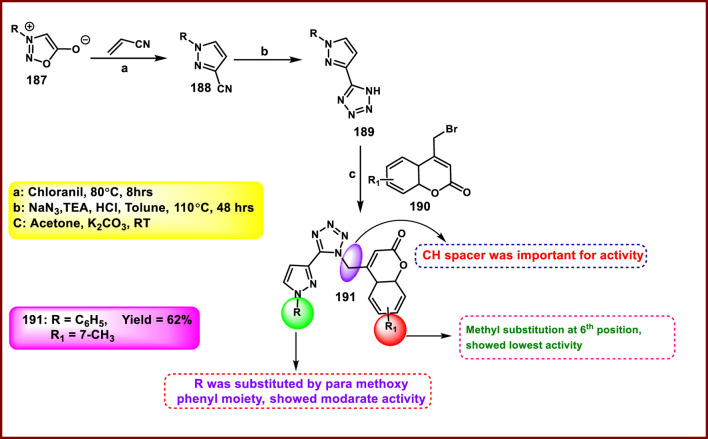
Synthesis and structure–activity relationship of 7-methyl-4-((5-(1-substituted-1*H*-pyrazol-3-yl)-1*H*-tetrazol-1-yl)methyl)-4*a*,8*a*-dihydro-2*H*-chromen-2-one.

Metre and coworkers developed pyrazole-fused tetrazole derivatives as promising dual-active anticancer and antifungal agents. In Scheme 35, the starting material *N*-arylsydnone **187** was converted to **188** in the presence of acrylonitrile and chloranil, followed by cyclization and nucleophilic substitution. Further, the reaction was carried out to yield the targeted compound **191** in the presence of substituted 4-bromomethyl coumarin **190**. All the synthesized compounds were subjected to an anticancer study, and results revealed that compound **191** showed IC_50_ values of 67.69 μg/mL and 27.85 μg/mL against the L929 and HCT116 cell lines, respectively. They tested its antifungal activity against *C. albicans,* and results demonstrated that compound **191** showed superior activity with MIC values of 4 μg/mL (Metre et al., 2024).

**SCHEME 36:**
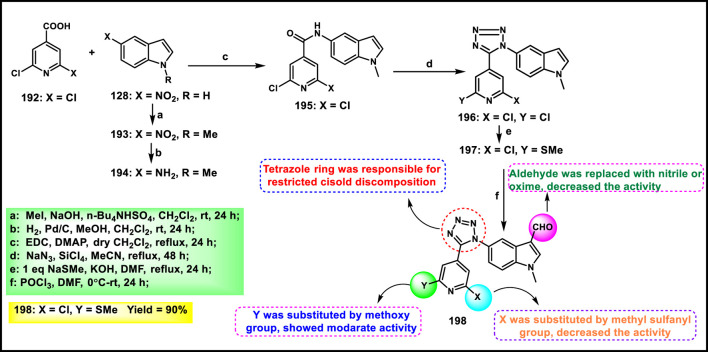
Synthesis of indole pyridine clubbed tetrazole derivatives as anticancer agents.

Yerga et al. designed and synthesized novel tetrazole derivatives targeting tubulin*. N*-methylation of 5-nitroindole **128** produced *N*-methyl-5-nitroindole **193** under basic conditions with phase transfer catalysis. Then, compound **193** was reduced to yield the corresponding amine **194**. In the next step, a coupling reaction was carried out between compound **194** and 2,6-dichloroisonicotinic acid **192** to yield amide intermediate **195**. The amide functionality in **195** was then transformed into a 1,5-disubstituted tetrazole ring using a tetrachlorosilane-azide system, resulting in compound **196**. The addition of a methylsulfanyl group to the pyridine ring was achieved via reaction with methanethiolate, giving compound **197**. Finally, a formyl group was introduced at the 3-position of the indole ring in compound **197** through a Vilsmeier–Haack reaction, resulting in the formation of the carbaldehyde derivative **198** (Scheme 36). First, they tested antiproliferative activity against the HeLa, MCF7, U87 MG, T98G, HepG2, HCT8, HT-29, and HEK-293 cell lines. The finding demonstrated that compound **198** showed the highest activity with IC_50_ values of 0.024 µM, 0.026 µM, 0.012 µM, 0.019 µM, 0.031 µM, 0.029 µM, 0.033 µM, and 2.27 µM, respectively. In further investigation, compounds were subjected to a tubulin polymerization inhibition assay, and results demonstrated that compound **198** showed the highest activity, with IC_50_ values of 0.8 µM. Flow cytometry studies revealed that the compound **198** targets tubulin and arrests cells at the G2/M phase, and thereafter undergoes apoptotic induction (Gallego-Yerga et al., 2023).

**SCHEME 37:**
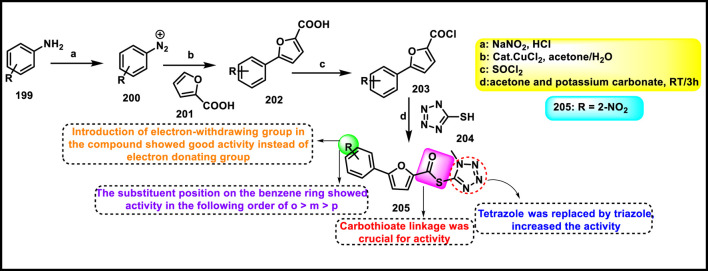
Synthesis and structure–activity relationship studies of *S-*(1-methyl-1*H*-tetrazol-5-yl) 5-(substituted phenyl)furan-2-carbothioate.

He et al. designed and synthesized furan-containing tetrazole derivatives as potent α-glucosidase inhibitors. The key intermediate **202** was synthesized using substituted aniline **199** and furoic acid **201** as starting substrates. Intermediate **202** reacts with thionyl chloride to yield the second intermediate **203** via an acylation reaction. Compound **204** was treated with **203** in basic media to produce the target compound **205** via nucleophilic substitution reaction (Scheme 37). All the synthesized compounds were subjected to an enzyme inhibition assay. The results showed that compound **205** demonstrated the highest inhibitory activity, with IC_50_ values of 4.6 µM and 78% inhibition at 50 µM concentrations against α-glucosidase kinase. The researchers conducted a cytotoxicity assay, revealing that compound **205** exhibited the highest activity against the HEK293, RAW264.7, and HepG2 cell lines, with percentage inhibitions of 22.3%, 33.9%, and 37.6%, respectively (He et al., 2023).

**SCHEME 38:**
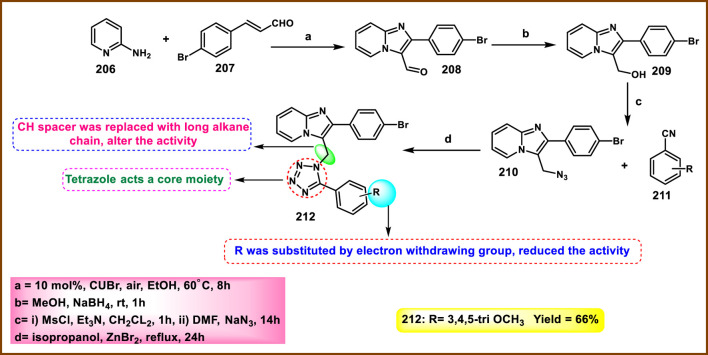
Synthesis of tetrazole-fused imidazopyridine compounds.

Vanam and Anireddy invented and synthesized tetrazole-fused imidazopyridine compounds as anticancer agents. In Scheme 38, a mixture of 2-aminopyridine **206** and 4-bromocinnamaldehyde **207** was stirred at 60 °C for 8 h to yield intermediate **208** through a copper-catalyzed cyclization reaction, followed by reduction in the presence of NaBH_4_ to afford compound **209**, and then mesylation and substitution by the azide ion to produce compound **210**. Finally, the cyclization reaction was performed between substituted benzonitrile **211** and azide **210** to produce the final tetrazole derivatives **212**. All the synthesized compounds were subjected to cytotoxicity studies against the MCF-7, A549, and MDA-MB-231 cell lines. The results revealed that compound **212** showed the highest activity with IC_50_ values of 1.66 μM, 1.90 μM, and 2.89 μM, whereas reference compound doxorubicin showed IC_50_ values of 3.12 μM, 2.10 μM, and 3.41 μM, respectively (Vanam and Anireddy, 2023).

**SCHEME 39:**
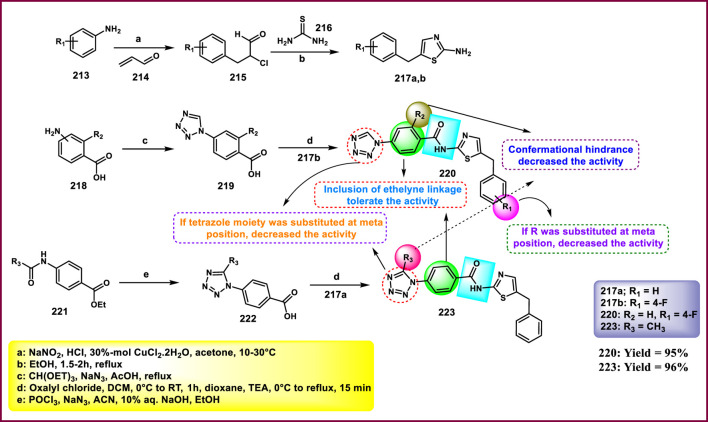
Synthesis of a tetrazole containing a benzylthiazol moiety.

Pokhodylo et al. developed a tetrazole containing a benzylthiazol moiety as a promising anti-leukemic agent. 2-amino-5-benzyl-1,3-thiazole derivatives **217a** and **217b** were synthesized via the Meerwein reaction of the arene diazonium chlorides, which are prepared from the corresponding aniline with acrolein, yielding *3*-aryl-2-chloropropanals, and the following cyclocondensation with thiourea. (1*H*-tetrazol-1-yl)benzoic acids **219** were obtained by the reaction of substituted 4-aminobenzoic acid **218** with triethyl orthoformate and sodium azide, followed by acylation of the reaction with compound **217b** in the presence of oxalyl chloride to yield compound **220**. The tetrazole acid **222** was prepared through a one-pot reaction involving amido-ester derivatives **221**, phosphorus oxychloride, and sodium azide. The present process was followed by hydrolysis of the ester moiety to form a carboxyl group and then acylation with compound **217a** in the presence of oxalyl chloride to yield compound **223** (Scheme 39). Synthesized compounds were subjected to an MTT assay for measurement of cell viability, and results revealed that compound **220** showed the highest activity against the K-562 cell line, with an IC_50_ value of 0.70 μM. Compound **223** showed promising activity against the HaCaT and J774.2 cell lines, with IC_50_ values of 6.00 μM and 3.61 μM, respectively. Compound **220** showed a significant effectiveness in inhibiting cell growth at nanomolar concentrations against both leukemia K-562 cells and melanoma UACC-62 cells, with IC_50_ values of 56.4 nM and 56.9 nM, respectively, as determined by the sulforhodamine B (SRB) assay. However, at nanomolar concentrations, compound **220** exhibited extreme cytotoxicity across a wide range of tumor cell lines, including K-562, NCI-H460, HCT-15, KM12, SW-620, LOX IMVI, M14, UACC-62, CAKI-1, and T47D. In the leukemic K-562 cells, the alkaline comet test identified that the compound **220** led to DNA single-strand damage. In K-562 cells treated with **220** at 70 nM, 1.1% of DNA was found in the tail of the comets, and 5.1% of DNA was found in the comet tails in cells treated with **220** at a 700 nM concentration. The reference drug, doxorubicin, caused less DNA damage than the derivative **220** (Pokhodylo et al., 2023).

**SCHEME 40:**
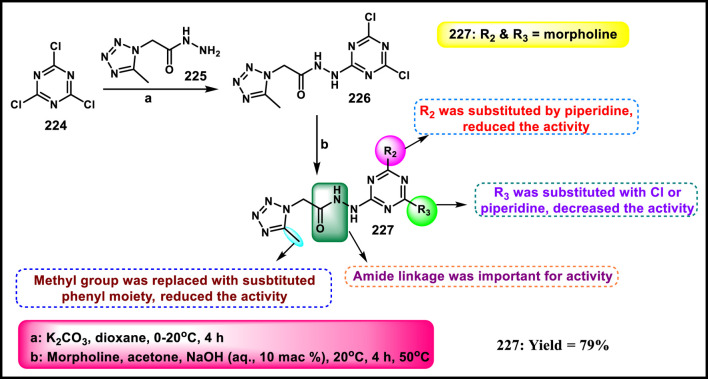
Synthesis of *N'*-(4,6-substituted-1,3,5-triazin-2-yl)-2-(5-methyl-1*H*-tetrazol-1-yl)acetohydrazide derivatives.

Mikolaichuk et al. developed tetrazole-fused morpholine derivatives as anticancer agents. In Scheme 40, cyanuric chloride **224** reacted with 5-methyltetrazol-1-ylacetic acid hydrazide **225** through a nucleophilic substitution reaction to produce compounds **226**, which have the tetrazole cycle connected to 1,3,5-triazine by an acetohydrazide linker. The resulting compound **226** was further used to synthesize targeted derivatives of morpholin-4-yl-1,3,5-triazines **227** under basic conditions. All the synthesized compounds were subjected to cytotoxicity studies against the Huh-7 and A549 tumor cell lines. Results demonstrated that compound **227** showed the highest inhibitory activity with a 49% and 51% inhibition percentage, respectively. The additional findings from the antioxidant analysis indicated that compound **227** did not interact with nitric oxide (NO) radicals and did not show any photoinduced hemolysis effect (Mikolaichuk et al., 2023).

**SCHEME 41:**
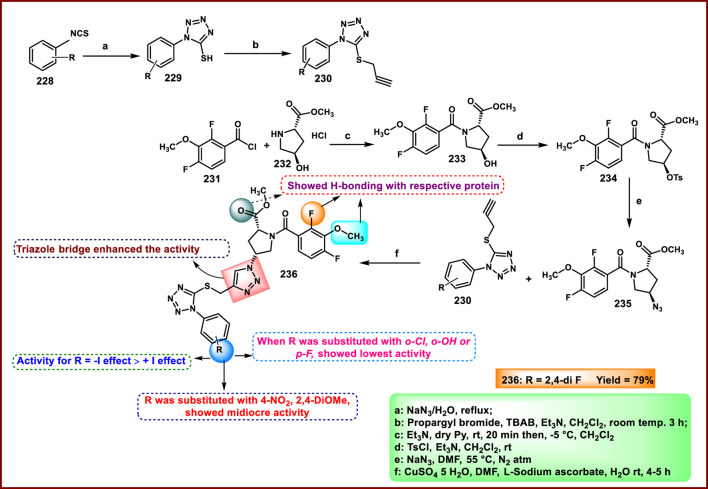
Synthesis of tetrazolo pyrrolidine analogs as potent anticancer agents.

Gandham and the group developed tetrazolo pyrrolidine analogs as potent anticancer agents. Intermediate **233** was synthesized by reaction with **231** and **232** in dichloromethane for 4 h through nucleophilic acyl substitution reaction, followed by the formation of a tosylate **234** from a hydroxyl group in the presence of 4-tolylsulfonyl chloride, which was further treated with sodium azide to provide azide intermediate **235** via SN2 reaction. Phenyl isothiocyanate **228** underwent a cyclization reaction in the presence of sodium azide to give 1-phenyl-1*H*-tetrazole-5-thiol **229**, followed by deprotonation and a nucleophilic substitution reaction to produce the derivative **230**. The synthesis of new tetrazolyl triazole pyrrolidine derivative **236** was accomplished via click chemistry of appropriate substituted tetrazole alkynes **230** with pyrrolidine azide **235** in the presence of L-sodium ascorbate and CuSO_4_ (Scheme 41). All the synthesized compounds were tested for anticancer activity against the HeLa, MCF-7, HCT-116, and HepG2 cell lines, and results showed that compound **236** exhibited the highest activity with IC_50_ values of 1.00 µM, 3.83 µM, 1.66 µM, and 1.92 µM, respectively. In comparison, the standard drug doxorubicin displayed lower activity than **236** with IC_50_ values of 2.34 µM, 3.02 µM, 1.96 µM, and 2.08 µM against the same cell lines (Gandham et al., 2023).

**SCHEME 42:**
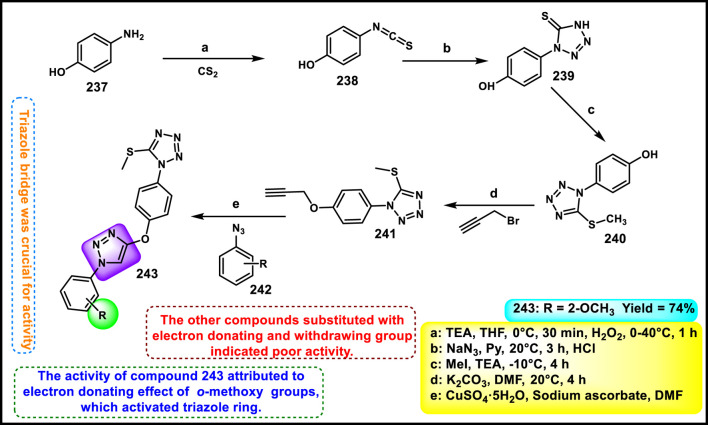
Synthesis of novel 1,2,3-triazole-linked tetrazole congeners.

Jarupula and coworkers developed novel 1,2,3-triazole-linked tetrazole congeners as a promising anticancer agent. In Scheme 42, 4-aminophenol **237** reacted with carbon disulfide in the presence of triethylamine in THF, followed by the addition of H_2_O_2_ at 40 °C to afford 4-isothiocyanatophenol **238**. Cyclization was carried out in the presence of sodium azide to afford 1-(4-hydroxyphenyl)-1,4-dihydro-5*H*-tetrazole-5-thione **239**, which was methylated and followed by a nucleophilic substitution reaction to produce intermediate **241**. Further reaction with substituted aryl azides **242** led to the formation of targeted derivatives **243** via a click reaction. All the synthesized compounds were screened for anticancer activity against the A549 and MCF-7 cell lines. Results revealed that compound **243** exhibited superior inhibitory activity, with IC_50_ values of 18.06 µM and 25.13 µM against the A549 and MCF-7 cell lines, respectively, compared to the standard drug doxorubicin, which had IC_50_ values of 20.48 µM and 27.18 µM against the same cell lines (Jarupula et al., 2023).

**SCHEME 43:**
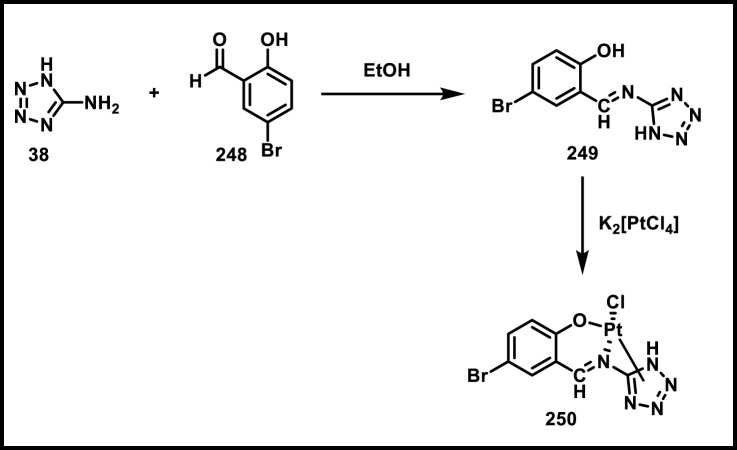
Synthesis of Pt(II) complexes of tetrazole derivatives.

Ulular and group-synthesized Ni(II) and Pt (II) complexes of tetrazole derivatives. 5-aminotetrazole **38** refluxed with 5-bromosalicyaldehyde **248** in ethanol to produce intermediate (*E*)-2-(((1*H*-tetrazol-5-yl)imino)methyl)-4-bromophenol **249**. A further reaction proceeds with K_2_ [PtCl_4_] to yield the target complex **250** (Scheme 43). The group screened the target derivatives for dual activities against cancer (binding properties of ct-DNA) and microbial strains. The binding properties of ct-DNA were investigated using ultraviolet-visible region spectroscopy and viscometry experiments. UV-region spectroscopy demonstrated that the molecules showed interaction with DNA, either electrostatic or binding to the minor/major grooves between base pairs of DNA and aromatic chromophore. Viscometry experiments suggested that all Schiff bases and complexes might also have intercalation-type interactions with DNA. The researchers also performed antimicrobial activity by the well-diffusion method. The complex **250** is the only ligand that was active against all microbial strains, both Gram-negative (*S. epidermis, S. aureus, and B. cereus*) and Gram-positive (*P. putida, E. aerogenes, S. typhi, E. coli, P. vulgaris, and C. albicans*). The complex showed a significant zone of inhibition with diameters of 22 mm, 23 mm, and 16 mm toward the respective Gram-negative strains and 26 mm, 14 mm, 20 mm, 19 mm, 10 mm, and 30 mm toward the respective Gram-positive strains mentioned above (Ulular et al., 2024).

**SCHEME 44:**
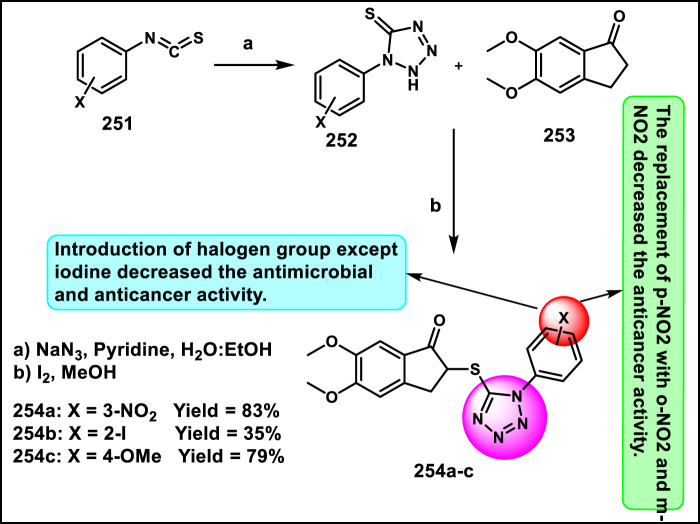
Synthesis of tetrazole-containing donepezil derivatives with multiple activities.

Disli et al. synthesized tetrazole-containing donepezil derivatives and investigated multiple activities, such as anticancer, antimicrobial, and antibiofilm (Scheme 44). Substituted phenyl isothiocyanate **251** underwent a cyclization reaction in the presence of sodium azide to give substituted 1-phenyl-1,2-dihydro-5*H*-tetrazole-5-thione **252**, which was further reacted with 5,6-dimethoxy-2,3-dihydro-1*H*-inden-1-one **253** to yield the target derivatives **254a-c** in the presence of iodine and methanol. The synthesized derivatives were screened against antibacterial (*E. coli ATCC 25,922* & *S. aureus ATCC 29,213*) and antifungal (*C. albicans ATCC 10,231*) strains. Compounds **254a** and **254b** showed excellent antimicrobial activity against *E. coli* (MIC = 0.1 and 0.2 mg/mL), *S. aureus* (MIC = 0.02 and 0.2 mg/mL), and *C. albicans* (MIC = 0.2 and 0.05 mg/mL) strains. The researcher performed an antibiofilm activity assay using the crystal violet method, and the results indicated that **254a** showed the highest biofilm inhibition rate of 90% and 98.74% in the *S. aureus* and *C. albicans* strains, respectively, at 0.2 mg/mL concentration. The anticancer activity was also examined using the MTT assay. All compounds were screened against the MDA-MB-231 cell line. The result showed that compounds showed moderate cytotoxicity, with IC_50_ values ranging from 475.36 to 589.18 µM. The researchers then tested a combination of doxorubicin and synthesized compounds and found that the compounds showed a synergistic effect. In combination, **254c** showed the highest activity against the MBA-MB-231 cell line, with the IC_50_ value of 1.14 µM. These results suggest that **254c** may enhance the anticancer potential of doxorubicin, which can be used as adjuvant combination chemotherapy targeting TNBC (Dişli et al., 2025).

## 4 Challenges and future perspectives

Tetrazole-based agents have enormous potential as antibacterial and anticancer therapeutics, but several obstacles prevent their clinical translation and extensive usage. Their adverse physicochemical profile is one of the main challenges. Because of their strong acidity and polarity, tetrazoles have poor membrane permeability and, as a result, low oral bioavailability. Their poor solubility, ineffective absorption, and occasionally high metabolism and excretion all limit their therapeutic efficacy, which reinforces this issue. Apart from pharmacokinetic problems, safety considerations are also important. Some tetrazole compounds have limited selectivity between impaired and healthy tissues, exhibiting cytotoxicity against normal cells. There may also be dangers of genotoxicity, mutagenicity, or hepatotoxicity, depending on the substitution pattern. In 2024, Kaur et al. reported indole-based tetrazole derivatives and screened for anti-breast cancer activity. Targeted derivatives displayed measurable cytotoxicity against the MCF-7 and MDA-MB-231 cell lines, but they also showed toxicity against normal human embryonic kidney cells (HEK-293), which indicates imitated selectivity (Kaur et al., 2024). In 2025, Vellaiyan et al. synthesized triazole–tetrazole conjugates that demonstrated potential antiproliferative effects on the HepG2 and HuH7 cancer cell lines while also damaging normal fibroblast cell lines at greater concentrations (Vellaiyan et al., 2025). Husain et al. reported copper (II) and cobalt (II) tetrazole complexes in 2023. These complexes exhibited robust DNA-binding and anticancer properties. However, their lack of selectivity resulted in considerable toxicity in normal cell models and oxidative stress in non-malignant tissues (Husain et al., 2023). Abdel-Rahman et al. (2025) synthesized benzimidazole-clubbed tetrazoles and screened them against the breast cancer cell line (MCF-7). The synthesized derivatives showed significant activity against the cancer cell line MCF-7, but also damaged the normal epithelial cells at the same concentrations. In antimicrobial applications, off-target toxicity may arise, and certain derivatives can disrupt the host microbiome, consequently diminishing therapeutic efficacy.

Structural factors also make things more challenging. Tetrazole rings often serve as bioisosteres for carboxylic acids and amides in drug design; however, this mimicry can lead to off-target interactions, increasing the risk of unintended side effects. For example, anticancer tetrazole derivatives may attack healthy cells that are dividing quickly, which can cause problems like bone marrow suppression, gastrointestinal toxicity, etc. Similarly, antimicrobial tetrazoles may be less effective against resistant strains or ineffective against multidrug-resistant pathogens. Chemical stability is another issue because some tetrazole derivatives can break down in water, become unstable in acidic or basic conditions, or break down in a way that depends on the pH. These problems make them less effective and shorten their shelf lives.

Even though there have been promising results *in vitro* and *in vivo*, only a small number of tetrazole derivatives have made it to clinical trials. Several strategies have been suggested to address these problems. Prodrug design may increase solubility and oral bioavailability. Combining tetrazoles with other pharmacophores in hybrid drug designs is a promising way to make drugs stronger and avoid resistance. In addition, computer-aided drug design (CADD) and other computational methods can help improve selectivity and reduce toxicity. Finally, rational structural changes can be made to fine-tune the balance between hydrophilicity and lipophilicity, which will improve the drug-like properties and overall therapeutic potential.

## 5 Conclusion

Tetrazoles, with their distinct nitrogen-rich heterocyclic structure, are still very promising for new drug development because of their wide range of biological activity, excellent physicochemical characteristics, and ease of synthetic accessibility. This study highlights the dual pharmacological potential, structure–activity correlations, and varied synthesis techniques of tetrazoles as antibacterial and anticancer drugs. The present study also summarizes the correlation between antimicrobial and anticancer activities, which will be beneficial for the design and development of multi-targeting candidates because anticancer and antimicrobial agents share their molecular targets and overlapping mechanisms, including DNA damage, protein synthesis inhibition, metabolic pathway interference, etc. Tetrazole’s potential in therapeutic development has been supported by recent research because several of the compounds discussed here have shown exceptional efficacy in antimicrobial and anticancer investigations. Certain compounds demonstrated broad-spectrum antibacterial activity with low MIC values, while others showed notable cytotoxicity against numerous cancer cell lines with IC_50_ values in the nanomolar range (Table 1). The increasing amount of data encourages more research and improvement of tetrazole templates, especially using hybridization techniques and structure-based design methodologies. According to these outcomes, tetrazole derivatives have much potential for the discovery and creation of novel therapeutic candidates.

**TABLE 1:** Summary of the most potent derivatives with MIC or IC_50_ and their respective strain or cell line.

S. No.	Compounds	MIC (µg/mL) or IC_50_ (µM)	Cell line or strain
1	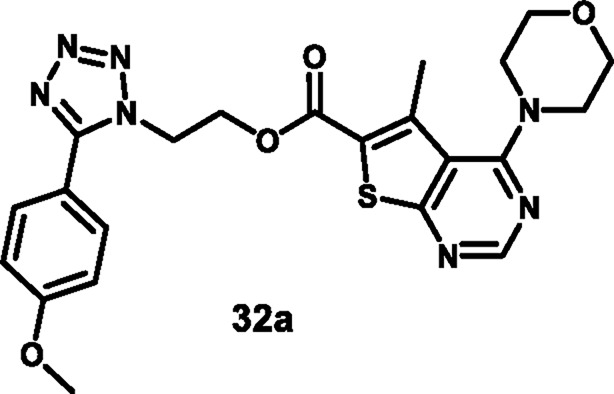	0.09 μg/mL 0.13 μg/mL	*E. coli* *P. vulgaris*
2	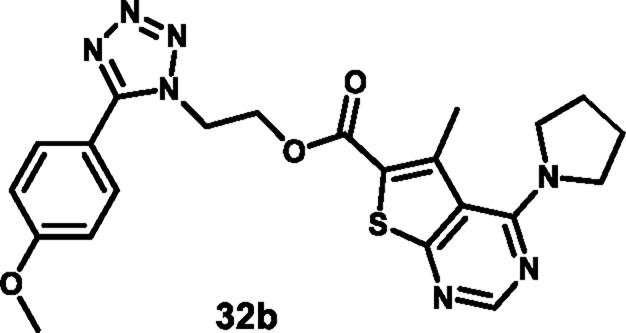	0.07 μg/mL 0.09 μg/mL 0.04 μg/mL 0.11 μg/mL	*B. subtilis* *E. coli* *A. niger* *R. oryzae*
3	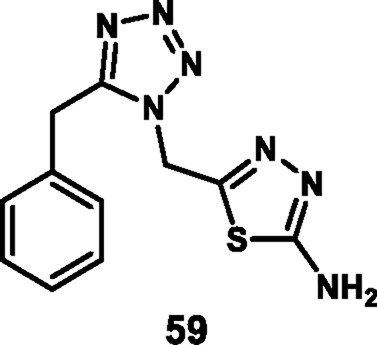	1.562 μg/mL	*S. aureus*
4	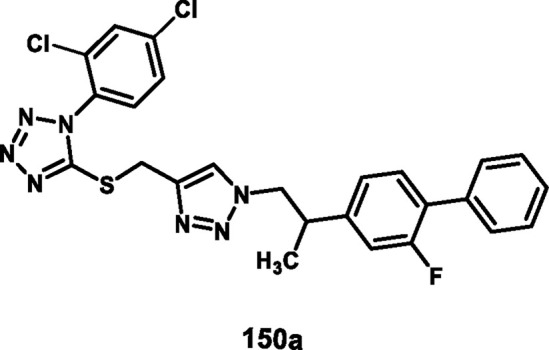	1.35 µM	MCF-7
5	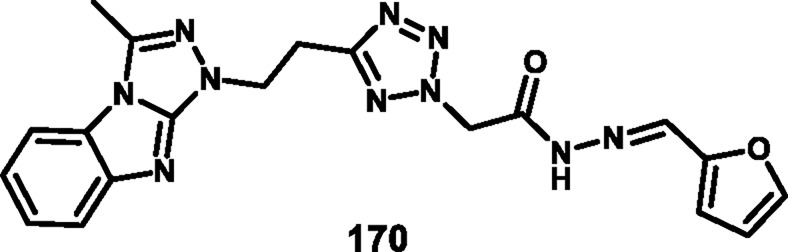	0.29 μM	MCF-7
6	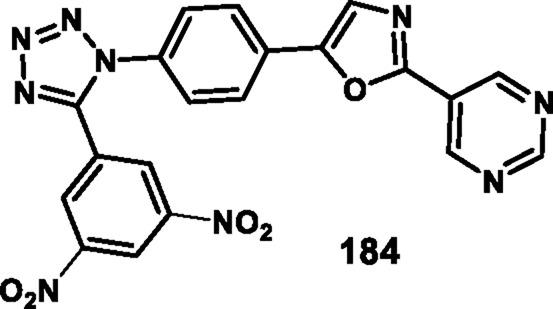	0.08 μM 0.04 μM 0.01 μM 0.12 μM	PC3 A549 MCF-7 DU-145
7	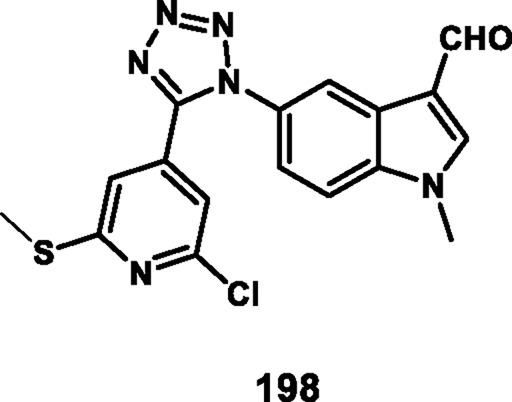	0.024 μM 0.026 μM 0.012 μM 0.019 μM 0.029 μM	HeLa MCF-7 U87 MG T98G HCT8
8	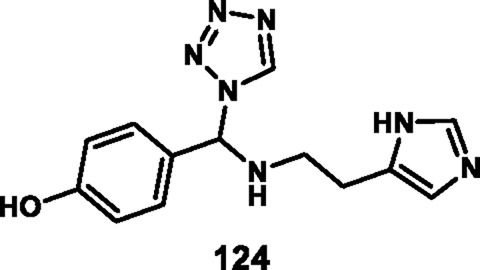	2 μg/mL 1 μg/mL GI50: 0.01 μM	*S. aureus* *M. audouinii* HeLa
9	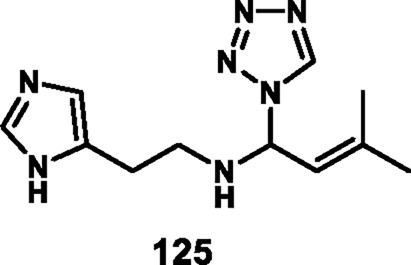	GI50: 0.02 μM GI50: 0.04 μM	HepG2 MCF-7
10	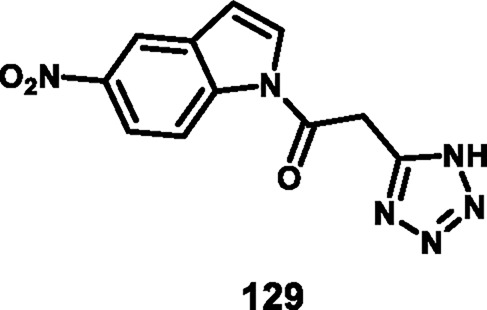	1.67 μg/mL 2.4 μM	*E. coli* MCF-7
